# Supplementary material for: Boosting the electron beam transmittance of field emission cathode using a self-charging gate
Source: Nat Commun. 2024 Jan 26;15:764. doi: 10.1038/s41467-024-45142-0 (PMC10817892; doi:10.1038/s41467-024-45142-0)
Supplement: Supplementary file 1 — Supplementary Information [file 41467_2024_45142_MOESM1_ESM.pdf]

# Supplementary Information

**Title**

Boosting the Electron Beam Transmittance of Field Emission Cathode Using a Self-Charging Gate

**Authors**

Dongyang Xiao[1,2], Huanhuan Du[1,2], Leimeng Sun[1*], Xiaochen Suo[2], Yurong Wang[2], Yili Zhang[2], Shaolin Zhang[2], Shuangyang Kuang[3], Fangjing Hu[2], Liangcheng Tu[2,4], Daren Yu[5] & Peiyi Song[2*]

**Affiliations**

[1]School of Optics and Electronic Information, Huazhong University of Science and Technology, Wuhan 430074, Hubei, China

[2]MOE Key Laboratory of Fundamental Physical Quantities Measurement & Hubei Key Laboratory of Gravitation and Quantum Physics, PGMF and School of Physics, Huazhong University of Science and Technology, Wuhan 430074, Hubei, China

[3]Hubei Key Laboratory of Plasma Chemistry and Advanced Materials, School of Materials Science and Engineering, Wuhan Institute of Technology, Wuhan 430205, Hubei, China

[4]MOE Key Laboratory of TianQin Mission, TianQin Research Center for Gravitational Physics & School of Physics and Astronomy, Frontiers Science Center for TianQin, Gravitational Wave Research Center of CNSA, Sun Yat-sen University (Zhuhai Campus), Zhuhai 519082, China

[5]Lab of Plasma Propulsion, Harbin Institute of Technology (HIT), Harbin 150001, China

**Contents**

Supplementary Note 1: The fabrication processes of the cathode. ................................3

Supplementary Note 2: The investigation of the stoichiometry of the $SiN_x$ electret. ....5

Supplementary Note 3: The patterning transfer technique for the cathode emitters. ..10

Supplementary Note 4: The material characterizations of the cathode. ......................13

Supplementary Note 5: The model of charge storage for the $SiN_x$/Au/Si gate. ..........15

Supplementary Note 6: The band structure of the $SiN_x$ electret. ................................18

Supplementary Note 7: The traps distributions of the $SiN_x$ electret. ...........................19

Supplementary Note 8: The hydrogen outgassing of the $SiN_x$ electret. ......................21

Supplementary Note 9: The model of charge decay of the $SiN_x$/Au/Si gate. ..............22

Supplementary Note 10: The electric field simulations of the cathode. ......................24

Supplementary Note 11: The effect of charging period on transmittance of cathode. 26

Supplementary Note 12: Study on charging saturation of the $SiN_x$ electret. ...............29

Supplementary Note 13: The Fowler-Nordheim plots of the cathode. ........................31

Supplementary Note 14: The effect of $SiN_x$ thickness on transmittance of cathode. ..32

Supplementary Note 15: Long-time measurement of the cathode. .............................35

Supplementary Note 16: The high current stability of the cathode. ............................36

Supplementary Note 17: The cathode performances upon the external stimuli. ..........38

Supplementary Note 18: The long-term current stability of the cathode. ....................51

**Supplementary Note 1: The fabrication processes of the cathode.**

The fabrication processes for the gate and the carbon nanotubes (CNTs) emitter are illustrated in Supplementary Fig. 1a and Supplementary Fig. 1b, respectively. The $SiN_x$/Au/Si gate was fabricated using a lithographic patterning process and film deposition techniques. Initially, the cleaned 300 μm Si wafer was patterned using the 13 μm AZ9260 photoresist layer, and the hexagonal gate array, with 228 μm side and 20 μm distance, was obtained after the deep reactive ion etching (DRIE) etching process and removing the photoresist layer in sequence. Subsequently, the Au/Ti (400/50 nm) as the metal electrode was deposited on the Si-based gate substrate using the EBE process, and a 1 μm $SiN_x$ layer was then deposited on the Au/Si gate substrate using the plasma-enhanced chemical vapor deposition (PECVD) process. Finally, the $SiN_x$/Au/Si gate was obtained successfully.

**Supplementary Fig. 1 Schematic diagram of the cathode**

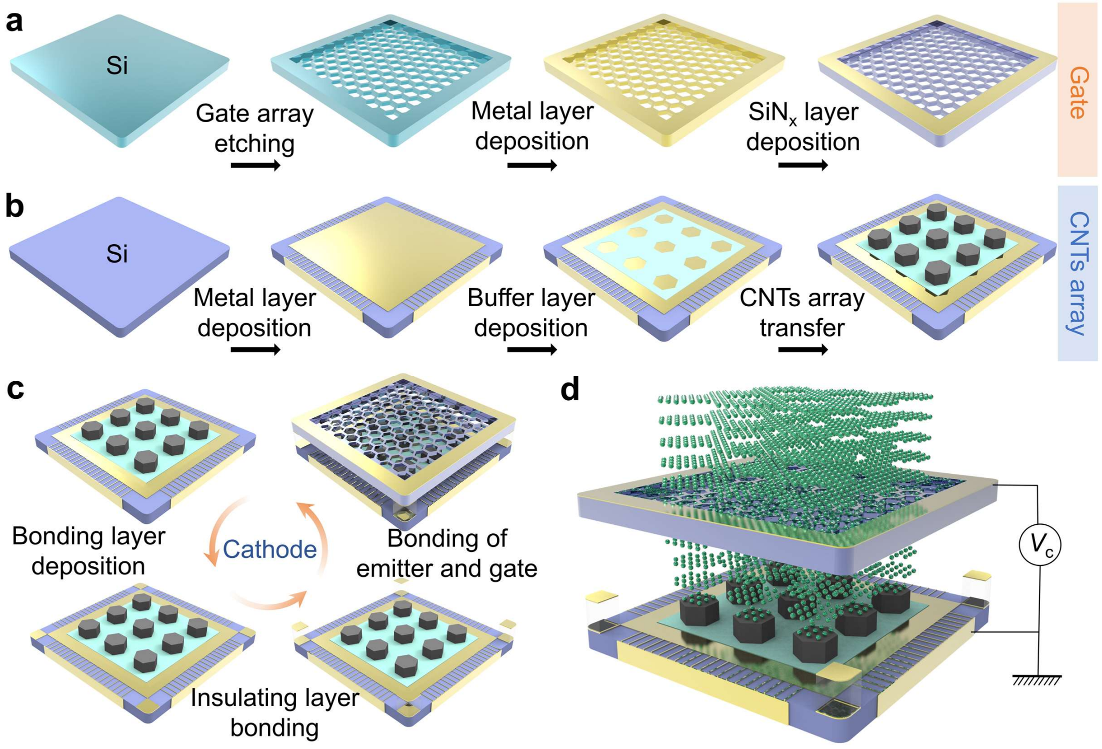


The fabrication processes of (**a**) gate and (**b**) carbon nanotubes (CNTs) array emitter, respectively. **c** The assembling process of the cathode. **d** The schematic of the operation process of the cathode under applied cathode emitter voltage $V_c$.

For the fabrication process of the cathode emitters, the CNTs emitter was first grown on 500 μm Si substrate after the deposition of $Al_2O_3$ and Fe as the buffer layer and catalyst, respectively, using an automated wafer-scale thermal chemical vapor deposition (TCVD) system. To enhance the adhesion between CNTs and substrate and reduce the shielding effect, a transfer process was used to bond the CNTs and target substrate. Second, the Au/Ni/Cr (500/20/50 nm) was deposited on the target Si wafer as a conducting layer using the electron beam evaporation (EBE) process, and the patterned buffer layer was then deposited on the Au/Si substrate using the PECVD process. Subsequently, the CNTs on the Si wafer were aligned and placed onto the target substrate with patterned Au/Ni/Cr (500/20/50 nm) on the surface. Under the treatment of 30 kPa pressure and temperature (300 °C) on both substrates simultaneously for 5 minutes, the patterned CNTs emitter on the target substrate was obtained after removing the original substrate.

Following the fabrication of the gate and CNTs emitter, the cathode was assembled utilizing micro-electromechanical systems (MEMS) bonding technology, as depicted in Supplementary Fig. 1c. Initially, a 400/50 nm thick layer of Au/Ti was deposited on both the top and bottom of the glass substrate to act as an insulating layer. Next, the thermocompression technique was employed to successfully bond the gate and CNTs emitter through the glass separator layer. When a voltage is supplied, the electrons are excited and pass through the gate under the focusing field, as demonstrated in Supplementary Fig. 1d.

**Supplementary Note 2: The investigation of the stoichiometry of the $SiN_x$ electret.**

It is important to note that during the utilization of PECVD for depositing $SiN_x$ films, deposition conditions such as gas flow rates, radio frequency (RF) power, substrate temperature, and others, can influence the material stoichiometry, consequently affecting the charging performance of the $SiN_x$ electrets. Extensive research has investigated the relationship among dielectric charging, material stoichiometry, and deposition parameters in PECVD $SiN_x$ films[1, 2].

To gain a deeper understanding of how the deposition parameters of PECVD $SiN_x$ films impact both material stoichiometry and the charging process. Various deposition parameters were employed in this study, involving alterations in reactive gas ratio, RF power, and substrate temperature. The samples under investigation comprise PECVD $SiN_x$ electrets with a thickness of 200 nm, deposited onto Si substrate with Au/Ti (200 nm/40 nm) layer through the PECVD (Oxford PlasmaPro 800 Stratum) process. Throughout the deposition process, reactive species such as silane ($SiH_4$) and ammonia ($NH_3$) were employed, with nitrogen ($N_2$) serving as the dilution gas. By employing alternating deposition using high frequency (HF) and low frequency (LF), the compressive stress generated at LF and the tensile stress generated at HF were effectively balanced, resulting in the fabrication of $SiN_x$ films with reduced compressive stress. In this process, the HF and LF are set at 13.5 MHz and 697 kHz respectively, with corresponding deposition times of 13 s and 7 s for one cycle, and the chamber pressure is set at 1000 mTorr. The primary reaction in the deposition process is illustrated as follows (Supplementary Equation 1.1):

$$SiH_4 + NH_3 \longrightarrow SiN_x + H_2 \tag{1.1}$$

As described above, $SiN_x$ films were deposited by altering various deposition parameters individually, including the gas ratio ($SiH_4/NH_3$), RF power, and substrate temperature. The investigated process parameters encompass the following ranges: gas ratio ($SiH_4/NH_3$) ranging from 0.45 to 1.36; RF power varying from 40 W to 50 W; and substrate temperatures ranging from 100 °C to 300 °C. All $SiN_x$ films were deposited while maintaining constant chamber pressure and RF frequencies. Supplementary

Table 1 presents a compilation of the diverse deposition parameters utilized in this study.

**Supplementary Table 1 Deposition parameters for $SiN_x$ electrets**

| $SiH_4$ (sccm) | $NH_3$ (sccm) | Total gas flow (sccm) | $SiH_4/NH_3$ gas ratio, *r* | RF power (W) | Substrate temperature (°C) |
|---|---|---|---|---|---|
| 15 | 11 | 811 | 1.36 | 50 | 100 |
| 15 | 11 | 811 | 1.36 | 45 | 100 |
| 15 | 11 | 811 | 1.36 | 40 | 100 |
| 15 | 11 | 811 | 1.36 | 50 | 300 |
| 15 | 11 | 811 | 1.36 | 45 | 300 |
| 15 | 11 | 811 | 1.36 | 40 | 300 |
| 10 | 11 | 811 | 0.91 | 50 | 100 |
| 5 | 11 | 811 | 0.45 | 50 | 100 |
| 10 | 11 | 811 | 0.91 | 50 | 300 |
| 5 | 11 | 811 | 0.45 | 50 | 300 |

After the completion of samples fabrication, the Fourier transform infrared spectroscopy (FT-IR) material characterization technique was employed to investigate the material stoichiometry of various $SiN_x$ films. FT-IR spectroscopy (Nicolet iS50R, Thermo Scientific, Inc.) was conducted to offer insights into the chemical bonds within the dielectric film and their alterations[1, 3]. IR spectra were acquired using a spectrometer in reflection mode at an angle of 70°, spanning the range of 400 $cm^{-1}$ to 4000 $cm^{-1}$.

Supplementary Fig. 2a depicts the FT-IR spectra of distinct $SiN_x$ films, deposited under varied RF power conditions, while maintaining a constant substrate temperature (100 °C) and $SiH_4/NH_3$ gas ratio ($r = 1.36$). Typically, three categories of bonds, namely Si–N, N–H, and Si–H, are observed. The peaks observed around 1031-1034 $cm^{-1}$ are attributed to the presence of the Si–N bond[1]. The peak at 2156 $cm^{-1}$ corresponds to the Si–H bond and peaks at 1188 $cm^{-1}$ and 3351 $cm^{-1}$ correspond to the N–H bond, indicating the incorporation of hydrogen into the film during growth from the source gases $SiH_4$ and $NH_3$. Supplementary Fig. 2b demonstrates the FT-IR spectra of distinct $SiN_x$ films, deposited under varied RF power conditions, while maintaining a constant substrate temperature (300 °C) and $SiH_4/NH_3$ gas ratio ($r = 1.36$). The peaks of the Si–N bond are observed around 1061-1064 $cm^{-1}$. The peak at 2171 $cm^{-1}$ corresponds to the Si–H bond and peaks at 1182 $cm^{-1}$ and 3345 $cm^{-1}$ correspond to the N–H bond. The

slight peaks position shift of the three bonds could potentially be attributed to variations in power during the $SiN_x$ films deposition process.

**Supplementary Fig. 2 The material stoichiometry of various $SiN_x$ films**

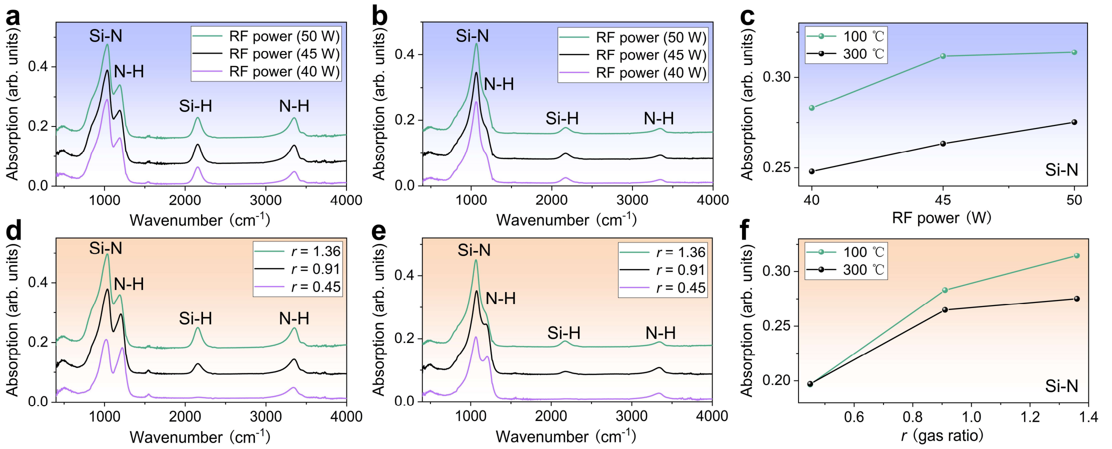


**a** Fourier transform infrared spectroscopy (FT-IR) of $SiN_x$ films fabricated under varying radio frequency (RF) powers. The green, black and purple curves represent the absorptions of different $SiN_x$ films when the RF power is 50 W, 45 W and 40 W, respectively, meanwhile maintaining a constant substrate temperature (100 °C) and $SiH_4/NH_3$ gas ratio ($r$ = 1.36). **b** FT-IR spectra of $SiN_x$ films fabricated under varying RF powers. The green, black and purple curves represent the absorptions of different $SiN_x$ films when the RF power is 50 W, 45 W and 40 W, respectively, meanwhile maintaining a constant substrate temperature (300 °C) and $SiH_4/NH_3$ gas ratio ($r$ = 1.36). **c** The absorptances of $SiN_x$ films versus RF power within different substrate temperatures. The green and black curves represent the absorptions of different $SiN_x$ films under various RF powers when the substrate temperature is 100 °C and 300 °C, respectively. **d** FT-IR spectra of $SiN_x$ films fabricated under different gas ratios. The green, black and purple curves represent the absorptions of different $SiN_x$ films when the gas ratio $r$ is 1.36, 0.91 and 0.45, respectively, meanwhile maintaining a constant substrate temperature (100 °C) and RF power (50 W). **e** FT-IR spectra of $SiN_x$ films fabricated under different gas ratios. The green, black and purple curves represent the absorptions of different $SiN_x$ films when the gas ratio $r$ is 1.36, 0.91 and 0.45, respectively, meanwhile maintaining a constant substrate temperature (300 °C) and RF power (50 W). **f** The absorptances of $SiN_x$ films versus gas ratio $r$ within different

substrate temperatures. The green and black curves represent the absorptions of different $SiN_x$ films under various gas ratios *r* when the substrate temperature is 100 °C and 300 °C, respectively.

In accordance with the analysis presented in Fig. 2b of the manuscript, the surface charging in $SiN_x$ electret is primarily attributed to amphoteric traps induced by dangling bonds, as elucidated by various studies. This model involves the charging of both electron and hole states, attributed to the presence of trivalent $Si^{3+}$ centers. Hence, our focus lies on the content of Si-N bond within the $SiN_x$ films, as it is positively correlated with $Si^{3+}$ centers. The absorptances of Si–N bond under various deposition temperatures, as extracted from FT-IR analysis, are depicted in Supplementary Fig. 2c. From the graph, it is evident that with the increase in RF power, there is an upward trend in the absorption peak of Si–N bond. Consequently, a higher RF power is more conducive to the formation of $Si^{3+}$ centers, thereby enhancing the charging performance of $SiN_x$ electrets.

Supplementary Fig. 2d-e illustrate the FT-IR spectra of different $SiN_x$ films, deposited at varying $SiH_4/NH_3$ gas ratios, while maintaining a constant RF power (50 W), at the substrate temperature of 100 °C and 300 °C, respectively. The absorptances of the Si–N bond under different deposition temperatures, as extracted from the FT-IR analysis, are illustrated in Supplementary Fig. 2f. Likewise, it's apparent that an increase in gas ratio corresponds to a rising trend in the absorption peak of the Si–N bond[1]. Consequently, a higher gas ratio promotes the formation of $Si^{3+}$ centers, thereby enhancing the charging performance of $SiN_x$ electrets. This phenomenon can be attributed to the increase in silicon content within the investigated $SiN_x$ films as the gas ratio increases. Furthermore, the FT-IR results from both Supplementary Fig. 2c and Supplementary Fig. 2f consistently indicate higher absorption peaks of the Si–N bond for films grown at lower temperature. This phenomenon can be attributed to the fact that low-temperature PECVD is more conducive to the formation of traps and dangling bonds within the films.

In conclusion, based on the findings presented above, to enhance the charging performance of the electret in this study, the growth conditions for $SiN_x$ are recommended to be set at a gas ratio of 1.36, an RF power of 50 W, and a substrate temperature of 100 °C.

**Supplementary Note 3: The patterning transfer technique for the cathode emitters.**

The strategy to suppress the field emitter shielding effect resides in the patterning design of CNTs, wherein the transformation of bulk CNTs configurations into patterned clusters, known as patterned CNTs clusters, is achieved. Due to the presence of edge effects, larger emitters exhibit stronger edge electric fields, resulting in a concentration of current emission primarily at the edge regions, consequently causing a shielding effect on current emission in the central area[4, 5]. Utilizing patterning techniques to reduce emitter size, to a certain extent, can suppress the impact of the shielding effect, thereby significantly increasing the cathode's current emission density.

Efforts have been made to choose the dimensions of the emitters array and suppress the shielding effect. For instance, a team led by M. Cole at the University of Cambridge has fabricated an arrayed emitter of single carbon nanotube (CNT) and conducted simulation analyses[6]. The electron emission capability becomes stronger as the spacing between the arrays increases from 1 μm to 10 μm. This conclusion has also been further confirmed, where research results indicate a significant change in current values as the array size increases from 70 μm to 1 mm[7]. In summary, the strength of the shielding effect is highly dependent on the spacing between the arrays.

By modeling and simulating arrayed one-dimensional materials, researchers have equivalently characterized the shielding effect as the ratio of the overall field enhancement to the field enhancement of a single element. A stronger shielding effect corresponds to a lower value of this ratio. The modeling and simulation results demonstrate that the strength of the shielding effect is determined by both the spacing between the arrays and the height of the emitter. This ratio can be expressed using the following equation[8]:

$$\frac{\beta}{\beta_0} = 1 - \exp(-a(\frac{b}{h})^c) \qquad 1.2$$

$\beta_0$ represents the field enhancement factor of a single CNT, $\beta$ denotes the average field enhancement factor of the cathode, a and c are constants, $b$ represents the array pitch, and $h$ represents the emitter height[9, 10]. As indicated by Supplementary Equation 1.2, the shielding effect is related to the ratio of the emitter array pitch and height. When the

value of *b/h* exceeds 2, the relative field enhancement effect saturates. In other words, when the ratio of array pitch to height exceeds 2, the emission performance of the cathode reaches its optimal state, with the influence of the shielding effect on electron emission capability minimized[11, 12]. This suggests that increasing the spacing between emitters can reduce the shielding effect, allowing for more uniform emission from multiple emitters and enhancing the overall field enhancement capability.

Based on the above analysis, it is evident that to suppress the impact of the shielding effect and achieve uniform field emission (FE), and it is desirable to have a ratio of the spacing to height of single CNT greater than 2. However, in practical applications, controlling the pitch of each CNT is challenging. Therefore, we have devised clustered CNTs array to alleviate the shielding effect between emitters. Similar to a single CNT array, clustered CNTs array also exhibits an optimal ratio of pitch to height that minimizes the shielding effect. Thus, we have designed CNTs clusters with different cluster sizes of 240 μm, 480 μm, and 720 μm, and employed CST simulations to investigate the variation of cathode current density with changes in the ratio of cluster pitch to height under the same voltage. As depicted in Supplementary Fig. 3a, when the ratio of CNTs array pitch to height exceeds 5, the current density reaches saturation, indicating the minimal shielding effect between the CNTs array.

By employing the described thermocompression process, taking into account the processing limitations, we fabricated three cathodes with different patterning sizes of emitters (240 μm, 480 μm, and 720 μm) and conducted FE testing. The ratio of array spacing to height was designed to be 6. From the test results shown in Supplementary Fig. 3b, it can be observed that the maximum current densities for the CNTs arrays with a side length of 240 μm, 480 μm, and 720 μm are 13.72 mA $cm^{-2}$, 5.97 mA $cm^{-2}$, and 3.34 mA $cm^{-2}$, respectively. This indicates that, under the same cathode voltage, the 240 μm unit possesses stronger electron emission capability, exhibiting the most significant suppression of the shielding effect. Therefore, all subsequent experimental designs were based on the 240 μm CNTs array.

**Supplementary Fig. 3 The shielding effect of the cathode emitter**

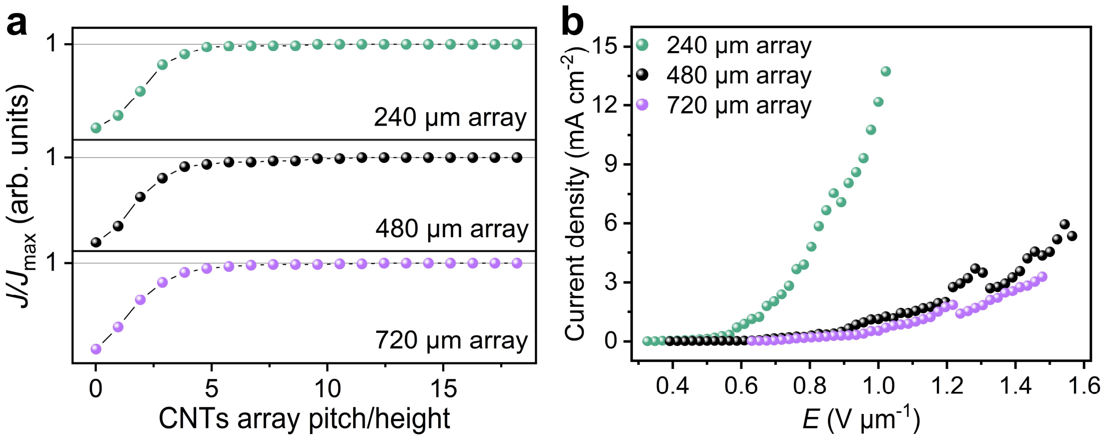


**a** Simulations for the variation of current density (*J*) as a function of the emitter pitch-to-height ratio, with three different side lengths for carbon nanotubes (CNTs) clusters: 240 mm, 480 μm, and 720 μm. The green, black and purple dots correspond to the normalized current densities when the patterning sizes of emitters are 240 μm, 480 μm and 720 μm, respectively. **b** The relationships between current densities and electric field strengths (*E*) for the CNTs arrays with a side length of 240 μm, 480 μm, and 720 μm under the same emitter pitch-to-height ratio. The green, black and purple dots correspond to the measured current densities when the patterning sizes of emitters are 240 μm, 480 μm and 720 μm, respectively.

**Supplementary Note 4: The material characterizations of the cathode.**

Supplementary Fig. 4a-d display the optical images of the $SiN_x$/Au/Si gate with 228 μm side and 20 μm distance, magnified for better visualization. Scanning electron microscopy (SEM) images in Supplementary Fig. 4e-f reveal the microscopic morphologies of the gate from different perspectives. To observe the section morphologies of the $SiN_x$/Au/Si gate, a high-precision DS-6 dicing saw was used to carefully cut the gate. The resulting section view can be found in Supplementary Fig. 4g, with a zoom-in view of the interfacial micro-morphologies between the Si substrate and the $SiN_x$/Au film shown in Supplementary Fig. 4h-i, with each layer clearly labeled. Supplementary Fig. 4j-m display the optical images of the fabricated cathode, which consists of a patterned CNTs emitter and $SiN_x$/Au/Si gate. SEM images in Supplementary Fig. 4n-o show the top and side views of the cathode, respectively. The gate-to-CNTs distance was set to 500 μm.

**Supplementary Fig. 4 Morphology characterizations of the cathode**

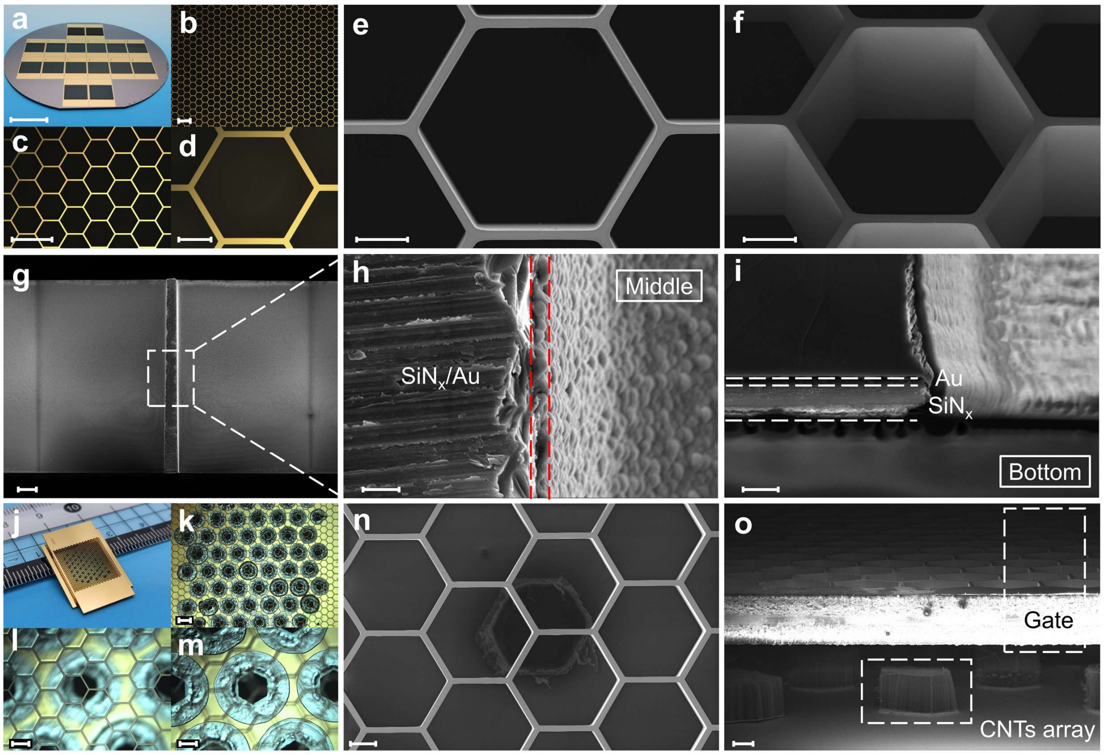


**a-d** Optical image of the $SiN_x$/Au/Si gate. Scale bars are 2 cm, 500 μm, 500 μm and 100 μm, respectively. **e, f** Top view of the scanning electron microscopy (SEM) images of the $SiN_x$/Au/Si gate. Scale bars, 100 μm. **g-i** Cross-sectional view of the SEM images of the $SiN_x$/Au/Si gate. Scale bars are 20 μm, 1 μm and 1 μm, respectively. **j** Physical diagram of the cathode. **k-m** Optical images of the cathode. Scale bars are 500 μm, 200 μm and 200 μm, respectively. **n** The top view and (**o**) side view of the cathode, including the gate and carbon nanotubes (CNTs) array. Scale bars, 100 μm.

**Supplementary Note 5: The model of charge storage for the $SiN_x$/Au/Si gate.**

As mentioned in previous work, electret materials possess the capability to store surface charges, space charges, and dipole charges[13]. Non-polar materials such as polytetrafluoroethylene (PTFE), fluorinated ethylene propylene polymer (FEP), silicon dioxide, and silicon nitride primarily store space charges, whereas polar materials like polyvinylidene fluoride (PVDF) exhibit predominant orientationally polarized dipole charges in addition to some space charges. Therefore, it is widely recognized that polar materials with polar molecules can generate dipole charges. In contrast, the non-polar $SiN_x$ material fabricated in this study does not exhibit dipole charges.

In addition, when employing electron beam (E-beam) irradiation for charging the electret materials, only space charges are deposited in the materials[13]. This is consistent with the method of gate charging in the cathode, which involves E-beam irradiation. Therefore, in the case of the $SiN_x$ electret investigated in this study, the stored charges primarily consist of space charges, and no dipole charges are present. To further illustrate the charge distributions within the $SiN_x$ after charging, Supplementary Fig. 5 depicts a schematic representation of the charge distributions before and after $SiN_x$-gate charging. As observed in the figure, after charging by E-beam irradiation, space charges (electrons) are distributed among different trap levels within the $SiN_x$ while compensating charges (positive charges) are induced on the Au electrode.

**Supplementary Fig. 5 The charge distributions of $SiN_x$/Au/Si gate**

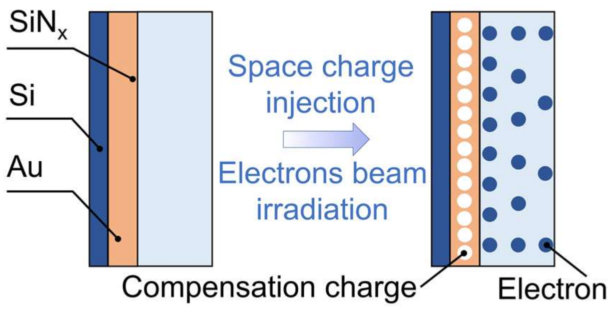


The charge distributions of $SiN_x$/Au/Si gate before and after electron beam (E-beam) irradiation charging.

Moreover, during the operation of the cathode, the external positive ions can be

attached at the $SiN_x$ film surface and affect the performance of the electret. The sources of external positive ions can be categorized into two main types: firstly, there is a minimal presence of positive ions in the external environment, which may migrate to the surface of the $SiN_x$-gate under the influence of external forces. These scarce positive ions on the surface of $SiN_x$ neutralize with the electrons present on the $SiN_x$ surface. However, the electrons emitted by the cathode are instantaneously injected into the $SiN_x$-gate. Even if some of these electrons on the $SiN_x$ are neutralized by positive ions, they are replenished by subsequent emitted electrons. Therefore, these exceedingly small quantities of positive ions do not impact the surface potential of $SiN_x$, and consequently, they do not affect the E-beam transmittance of the cathode.

Secondly, the cathode frequently serves as the neutralizer in electric propulsion systems, supplying electrons to neutralize positive ions or positively charged droplets, thereby preventing the spacecraft from accumulating charge. When the cathode and thruster work in coordination, their positioning relationship, as shown in Supplementary Fig. 6a, involves a certain angle. This is to prevent positively charged ions and droplets emitted by the thruster from splashing onto the surface of the cathode due to the electric field acceleration, which could affect the cathode's FE performances.

**Supplementary Fig. 6 The collaboration between cathode and thruster**

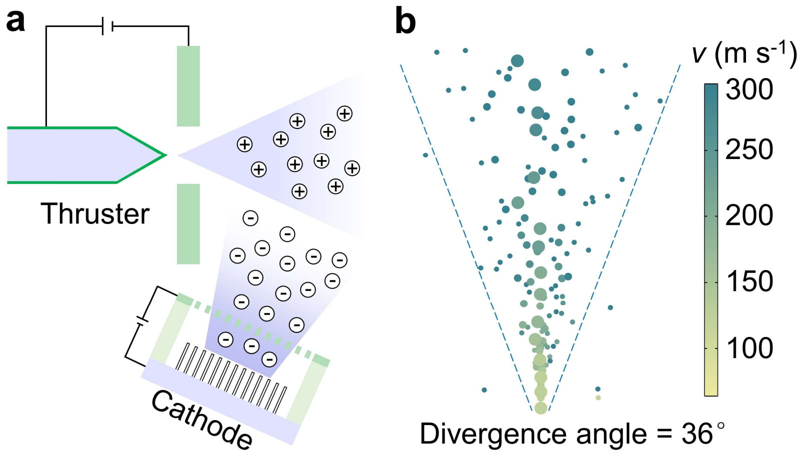


**a** Positional relationship of thruster and cathode during synergistic operation. **b** Simulation of the trajectories of charged ions and droplets emitted by the thruster, *v* represents the velocity of ions and droplets.

More specifically, the thruster emits a plume with a small divergence angle and high velocity in our simulation (Supplementary Fig. 6b), and the research findings from other studies have also confirmed this phenomenon[14, 15]. Therefore, under the influence of the electric field acceleration, positively charged ions or droplets are unlikely to splash onto the surface of the $SiN_x$-gate electrode. In other words, this setup does not impact the charging performance of the $SiN_x$-gate electrode.

According to the research conducted by V. Kleshch et al., when the thruster and cathode operate together, the electrons emitted by the cathode are attracted and deviate from their original trajectory towards the positively charged ions emitted by the thruster[16]. This ensures effective neutralization between electrons and ions. Furthermore, even if some positively charged ions have a chance to splash onto the area above the cathode, these ions are neutralized by the electrons emitted by the cathode, thus not affecting the high transmittance characteristics of the $SiN_x$-gate. In summary, positively charged ions under electric field acceleration do not impact the charging performance of the $SiN_x$-gate.

**Supplementary Note 6: The band structure of the $SiN_x$ electret.**

During the fabrication process of $SiN_x$, factors such as doping and oxidation can introduce defects and impurities, leading to the formation of defect states within the band structure. Additionally, the highly disordered structure and composition of amorphous $SiN_x$ result in the presence of localized states within the bandgap, known as band-tails[17]. Extensive studies have been conducted on the band-tails of amorphous $SiN_x$, revealing that these band-tails within the bandgap can form a continuous energy distribution between the conduction and valence bands[17, 18]. According to the amphoteric traps theory mentioned above, electrons are primarily captured by trap levels associated with dangling bonds in $SiN_x$, leading to the formation of a negative surface potential on the $SiN_x$ surface. This energy level diagram helps to provide a more detailed understanding of the electron trapping mechanism in the $SiN_x$ electret of the $SiN_x$/Au/Si gate structure.

**Supplementary Note 7: The traps distributions of the $SiN_x$ electret.**

As reported by U. Zaghloul et al., gas ratio, RF power, and substrate temperature have been found to influence the trap distributions in $SiN_x$, thereby affecting the charging and discharging processes[1]. The reactive gas ratio has been observed to have a more significant impact on the stoichiometry of $SiN_x$ material compared to the effects of RF power or substrate temperature from the FT-IR results. As the $SiH_4/NH_3$ gas ratio increases from 0.15 to 0.45, the injected charge density and relaxation time constant gradually decrease. However, for gas ratios of 0.6 and 0.8, the decrease becomes more pronounced. This trend can be attributed to the increased silicon content in the $SiN_x$ film, as confirmed by FT-IR data. The higher silicon content results in a higher leakage current, a greater concentration of defects, and an increased number of charge-trapping centers. Consequently, the charge redistribution paths become more extensive with higher silicon content in the $SiN_x$ film, leading to a larger redistribution current and a smaller charge density at the dielectric surface[1].

In summary, the variations in gas ratio, RF power, and substrate temperature can impact the trap level distributions in $SiN_x$ film, thereby effecting the charging performances of the gate based on the $SiN_x$ film. As shown in Supplementary Fig. 7, with higher defect concentrations, there are more charge-trapping centers, resulting in multiple charge redistribution paths within the $SiN_x$ material. As a consequence, the charge density at a specific location decreases due to the increased availability of charge pathways within the $SiN_x$ film.

**Supplementary Fig. 7 The trap level distributions of $SiN_x$**

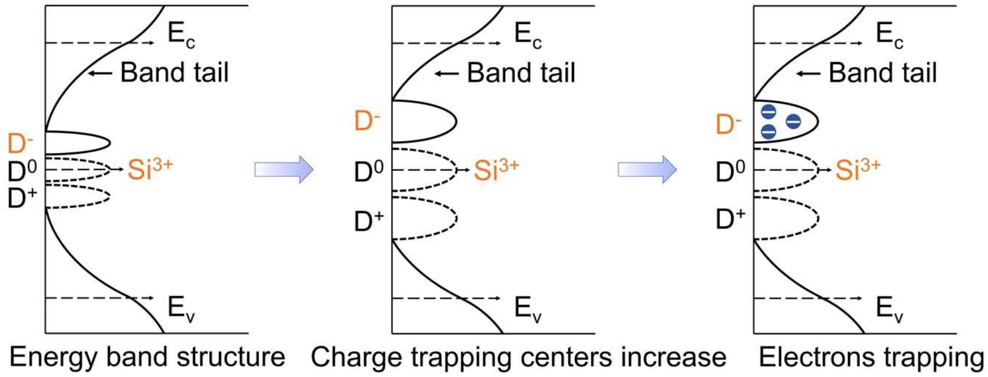


When a single electron is captured by a dangling bond, the charge state is neutral ($D^0$). The negative ($D^-$) state occurs when two electrons are attached to the dangling bond. Conversely, in the absence of electron bonding, a hole attaches to the dangling bond, resulting in a positive ($D^+$) state. The band structures include the defect states ($Si^{3+}$), band-tails, conduction band ($E_c$) and valence band ($E_v$).

**Supplementary Note 8: The hydrogen outgassing of the $SiN_x$ electret.**

From Supplementary Equation 1.1, a certain quantity of hydrogen is incorporated into the $SiN_x$ matrix during the PECVD process. According to the research findings by A. Picciotto et al., the hydrogen content in $SiN_x$ materials can indeed influence the material's stress levels[19]. The correlation between the annealing temperature of $SiN_x$ and the resulting film stress has been investigated, and the observed effect is likely a result of the outgassing of hydrogen, which is typically present in PECVD $SiN_x$ film and originates from the reaction between $SiH_4$ and $NH_3$ within the chamber. This outgassing leads to a reorganization of the nitride structure formed by both HF and LF layers, resulting in a change in stress towards positive values. Furthermore, this outgassing phenomenon becomes more pronounced with increasing annealing temperature. However, in contrast to thermionic cathodes, CNTs-based cathode can operate at room temperature[20, 21]. Therefore, the impact of annealing temperature on the stress of $SiN_x$-gate is not considered in practical processes.

Furthermore, based on the elemental composition characterization results of $SiN_x$ presented in Fig. 2d of the manuscript, it is evident that the hydrogen content within the $SiN_x$ electret is low. The primary constituents include Si, N, C, and O. Therefore, in practical applications of $SiN_x$ electret, the impact of hydrogen outgassing on film performances is minimal.

**Supplementary Note 9: The model of charge decay of the $SiN_x$/Au/Si gate.**

As shown in Fig. 2g of the manuscript, the gate surface potential ($V_s$) based on $SiN_x$ electret is approximately -60 V after E-beam irradiation for 12 hours and decreases by around 40% within a week, which is attributed to the de-trapping of electrons from the electret materials. As illustrated in Supplementary Fig. 8, the electrons emitted from the CNTs emitter can be trapped by the $SiN_x$/Au/Si gate due to the charge storage performance, and the initial $V_s$ of the gate is $V_0$ after charge injecting. The research of surface potential decay of $SiN_x$ has been demonstrated in previous work[22-24]. U. Zaghloul et al. revealed the correlation between relative humidity and the charging and discharging processes of $SiN_x$ material in both air and nitrogen environments[22]. As relative humidity increases, the injected charge density rises while the discharge process accelerates. Several factors contribute to the increase in injected charge density, including the conductivity of the adsorbed water film, the presence of surface charges on the dielectric surface, and the less confined electric field distribution between the tip and sample surface as the adsorbed water layer expands under higher relative humidity levels. Additionally, the relaxation time decreases for both air and nitrogen with increasing humidity. This decrease is primarily attributed to the faster neutralization of surface charges with the external medium and the increased conductivity of the adsorbed water film over the $SiN_x$ surface.

**Supplementary Fig. 8 The mechanism analysis of the surface potential**

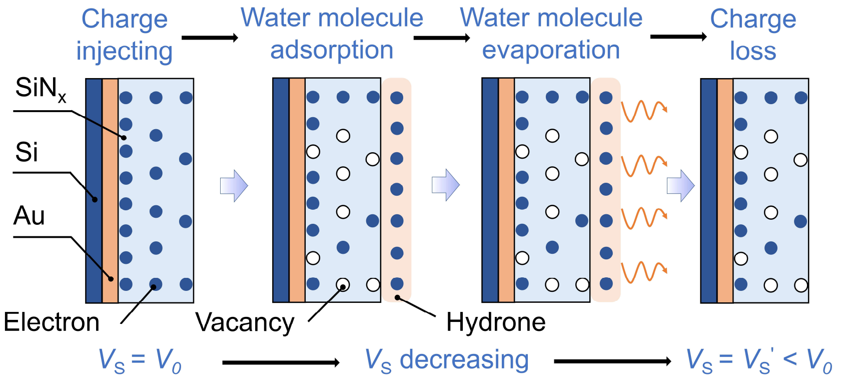


The mechanism analysis of the surface potential ($V_s$) dependence on the time. $V_0$ represents the initial surface potential of the $SiN_x$/Au/Si gate after charge injecting, $V_s$' represents the surface potential of the $SiN_x$/Au/Si gate after discharging for a week.

Hence, as the ambient humidity around the gate rises, a conductive water layer forms on the gate surface due to chemical and physical adsorptions. The increases in surface conductance and water molecule evaporation accelerate the charge decay of the $SiN_x$ electret surface[25], resulting in the decrease in the $V_s$ over time. After the conduction and water molecule evaporation processes, the electrons trapped in deeper positions of $SiN_x$ electret are retained instead of being released, resulting in a nearly 60% retention ($V_s'$) of the initial potential ($V_s$) level after discharging for a week[26]. As a caveat, the $V_s$ of $SiN_x$ electret will remain at a relatively constant level rather than decrease over time under cathode operation due to continuous electrons injection and low humidity of the vacuum environment. This is beneficial for the stability of charge storage and thus providing the high E-beam transmittance.

**Supplementary Note 10: The electric field simulations of the cathode.**

The three-dimensional simulation models are presented in Supplementary Fig. 9a. Here, the thickness, side length, and spacing of the gate unit are 300 μm, 228 μm, and 20 μm, respectively. Similarly, the thickness, side length, and spacing of the CNTs emitter are 110 μm, 240 μm, and 900 μm, respectively. The distance between the gate and the CNTs emitter is set to 500 μm, and the electric potentials of $V_1$, $V_c$, $V_g$, and $V_a$ denote the potentials of the $SiN_x$ electret, cathode substrate, Au electrode, and anode, respectively. Supplementary Fig. 9b shows the average electric field strength of the CNTs emitter surface when $V_1$, $V_c$, $V_g$, and $V_a$ are set to 0 V, -500 V, 0 V, and 0 V, respectively, which is approximately $2.35\times10^6$ V $m^{-1}$. When $V_1$ is set to -60 V and other electric potentials of the cathode are set to the same value as mentioned above, the average surface electric field strength of the CNTs emitter is approximately $2.07\times10^6$ V $m^{-1}$. Consequently, the addition of $SiN_x$ results in an 11.91% reduction in the surface electric field strength of the cathode, as compared to the case without $SiN_x$. The slight reduction in field strength can be attributed to the negative potential applied to the $SiN_x$ electret.

**Supplementary Fig. 9 Simulations of the cathode with and without $SiN_x$ electret**

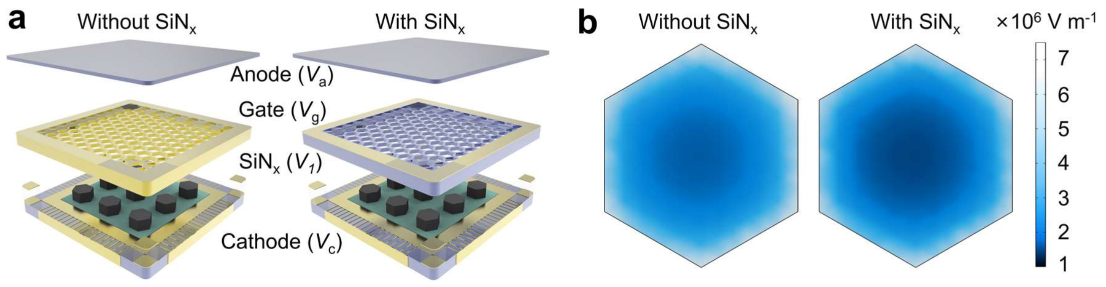


**a** Schematic of the simulation models of the cathode, the electric potentials of $V_1$, $V_c$, $V_g$, and $V_a$ denote the potentials of the $SiN_x$ electret, cathode substrate, Au electrode, and anode, respectively. **b** The electric field distributions of the surface of the carbon nanotubes (CNTs) emitter.

The slight reduction in the surface electric field strength of the CNTs emitter does indeed contribute to a decrease in the cathode's current emission density to some extent. Hence, to further enhance the current density of the cathode, we performed interface

modification on the emitter material. The modified CNTs emitter exhibited significantly increased current density. As shown in the test results of Supplementary Fig. 10, the maximum current density of the modified cathode is 17.54 mA $cm^{-2}$, compared to 1.27 mA $cm^{-2}$ before modification, representing an approximately 13-fold increase in current density. Even at high current densities, the average E-beam transmittance of the cathode's gate remains above 96%, providing further evidence of the feasibility of our cathode design at high current density operation.

**Supplementary Fig. 10 The current densities of the cathodes**

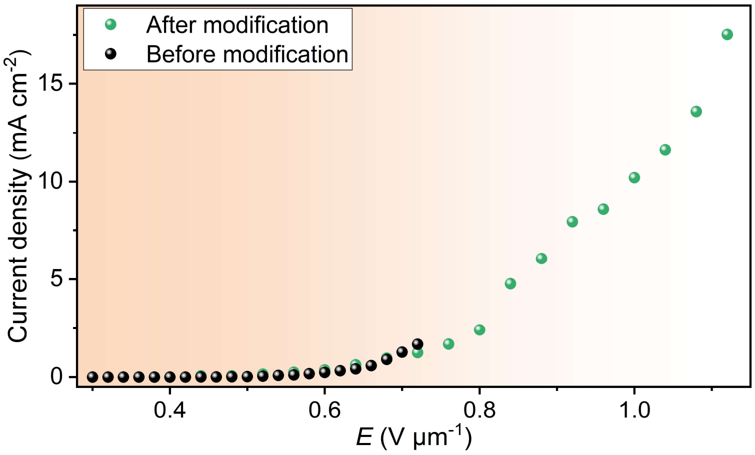


The relationships between current densities and electric field strengths ($E$) of proposed cathode before and after modification of carbon nanotubes (CNTs) emitter. The green and black dots correspond to the measured cathode current densities when the emitters are modified and unmodified, respectively.

**Supplementary Note 11: The effect of charging period on transmittance of cathode.**

Supplementary Fig. 11a illustrates the schematic diagram of the cathode emission current measurement system. As depicted in the diagram, when a negative high-voltage source (Wisman, DL5N300) is applied as the voltage input to the cathode emitter and the $SiN_x$-gate, the current generated by the emitter is successively intercepted and collected by the $SiN_x$-gate and the anode. The current signals collected by the $SiN_x$-gate and anode are initially output through the high-voltage flange of the vacuum system to the electrometer (Keithley, 6514). The electrometer can accommodate current signals ranging from 0 to 20 mA. Subsequently, the electrometer transmits the collected current signals to the data acquisition (DAQ) system (including the NI BNC-2121 Connector and the NI PCI-6250 Multifunction Acquisition Module). The DAQ system is capable of converting analog current signals into digital signals for reading and storage, with a sampling rate that can reach up to ~kHz. Ultimately, we can monitor the collected current data on the computer. Supplementary Fig. 11b provides a visual representation of the cathode emission current measurement system.

**Supplementary Fig. 11 The current measurement system of the cathode**

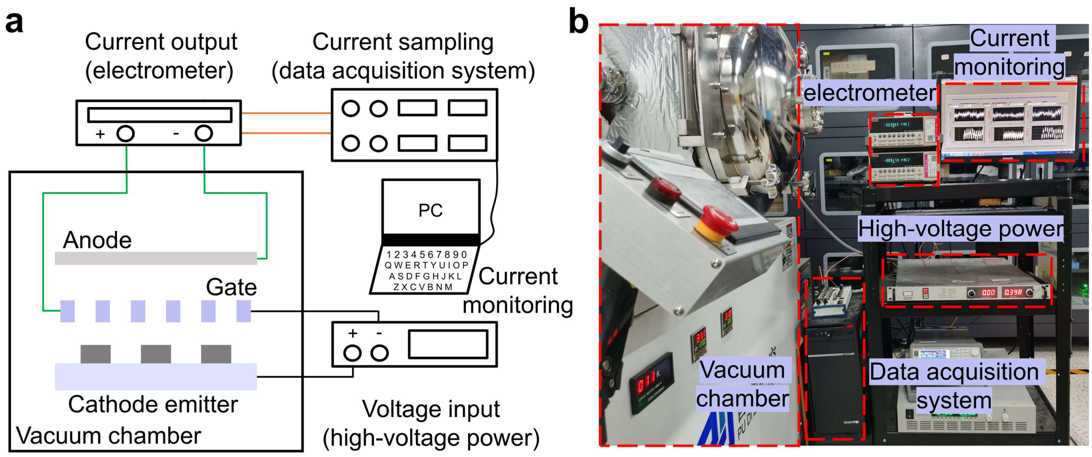


**a** The schematic diagram of the cathode emission current measurement system. **b** The image of the cathode emission current measurement system.

To further investigate the impact of the charging period on the E-beam transmittance of the cathode, we designed the following experiments to detect the current using the high-sampling-rate (2000 Hz) acquisition system immediately after

the cathode's initiation. We conducted measurements using different $SiN_x$-gate samples to assess the changes in cathode and anode currents during the cathode startup process, ranging from 0 to ~125 μA, ~200 μA, ~300 μA, and ~450 μA (corresponding to Supplementary Fig. 12a-d, respectively), thereby calculating the variations in cathode transmittance.

**Supplementary Fig. 12 The charging period of $SiN_x$-gate**

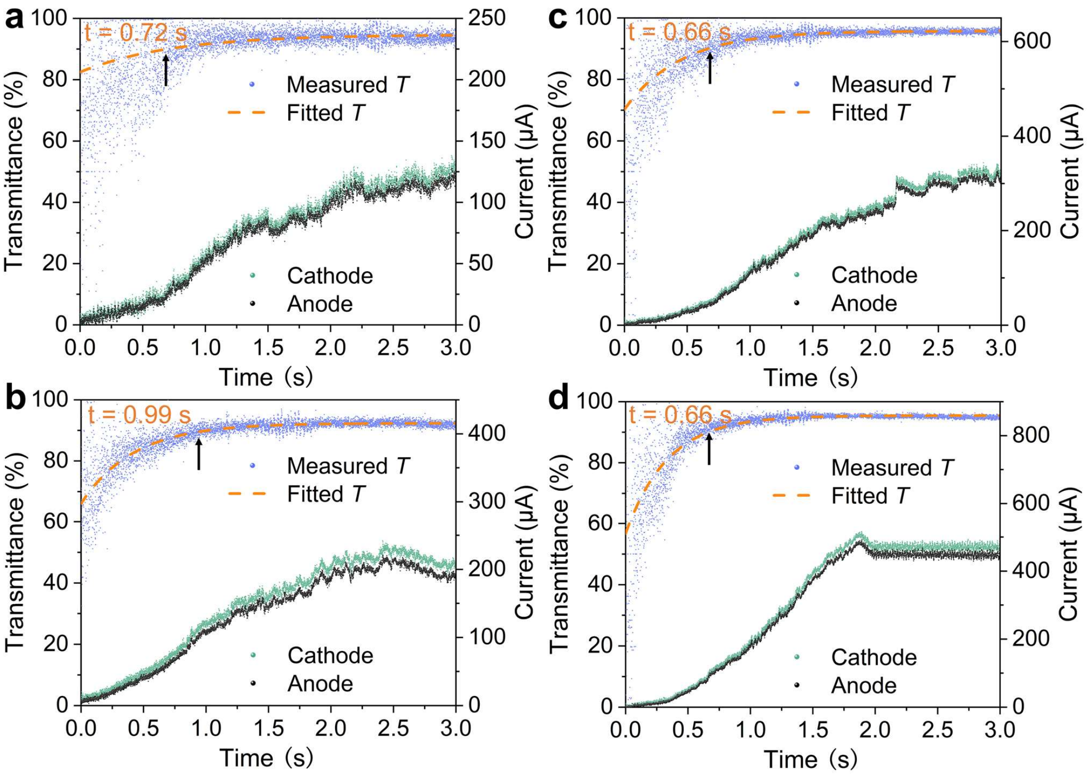


As the cathode current increases from 0 to (**a**) ~125 μA, (**b**) ~200 μA, (**c**) ~300 μA, and (**d**) ~450 μA, the emission current and transmittance as functions of the turn-on time of cathode, *T* represents the electron beam (E-beam) transmittance. The blue, green, and black dots correspond to the measured E-beam transmittance, cathode current, and anode current, respectively. Meanwhile, the yellow dashed line represents the fitted E-beam transmittances.

The test results indicate that at the moment of cathode startup, the emission current is minimal, resulting in a low and unstable E-beam transmittance through the corresponding $SiN_x$-gate. As the cathode emission current gradually increases, the charging of the $SiN_x$-gate saturates over time, leading to a gradual increase in the E-

beam transmittance through the gate, eventually reaching a stable state. The measured and fitted curves of E-beam transmittance in these figures demonstrate that although the final emission currents attained by the cathode differ, the time required for the gate E-beam transmittance to exceed 90% is consistently less than 1 s. Furthermore, it takes less than 1.5 s for the E-beam transmittance through the gate to reach its maximum value. This not only demonstrates the $SiN_x$-gate's exceptional electrons trapping performance and remarkably swift charging response time, but also indicates that CNTs cathode based on $SiN_x$-gate can be employed in cathode applications necessitating rapid response time.

**Supplementary Note 12: Study on charging saturation of the $SiN_x$ electret.**

Due to the limited trap density of electret materials, after $SiN_x$ electret reaches charging saturation, the surface potential of the $SiN_x$-gate will cease to change with increasing charging time[27]. Similarly, upon achieving charging saturation in the $SiN_x$-gate, even if we increase the negative high voltage on the cathode to increase the cathode emission current/current density, theoretically, the surface potential of the $SiN_x$-gate will not change. This is because, at this stage, the electrons trapped within the $SiN_x$ have reached saturation, leading to a stable surface potential (Supplementary Fig. 13a). To further validate this, we first charged the $SiN_x$ electret material. Once saturation was achieved, we gradually increased the charging voltage and measured the resulting changes in surface potential. Experimental results indicate that after charging saturation, the impact of voltage on the surface potential of $SiN_x$ is minimal (Supplementary Fig. 13b).

**Supplementary Fig. 13 The charging saturation of the $SiN_x$ electret**

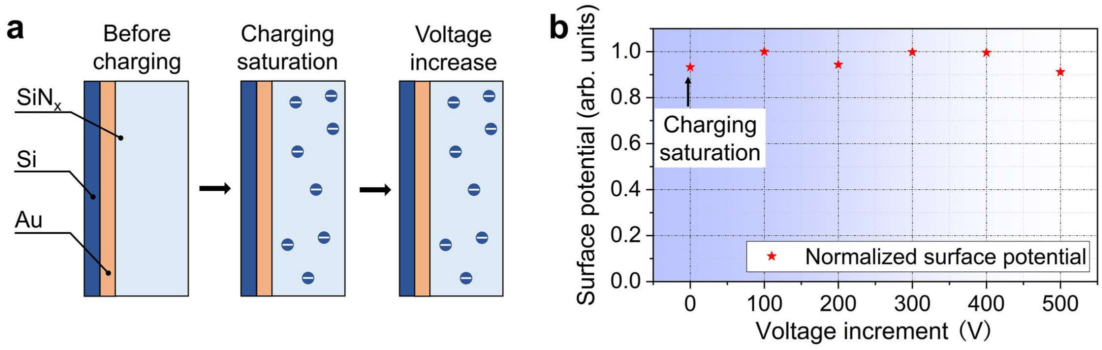


**a** The illustration for charging saturation of $SiN_x$ electret. **b** Variation of the normalized surface potential of $SiN_x$ electret with charging voltage curve after the charging saturation of the $SiN_x$ electret.

Based on the above analysis, it can be observed that after saturation of the charging of the $SiN_x$-gate, increasing the cathode voltage does not result in any significant change in the surface potential of the gate. Building upon this foundation, while maintaining the surface potential of the $SiN_x$-gate constant, we gradually increased the cathode emitter potential to investigate changes in the trajectories of emitted electrons from the cathode. As depicted in Supplementary Fig. 14a-c, it can be observed that with the $SiN_x$-gate potential unchanged, as the cathode emitter potential increases from -500 V to -

700 V, the $SiN_x$-gate is capable of achieving E-beam focusing, thereby achieving a high E-beam transmittance. Though the charging performances of dielectric materials can be influenced by the fabrication process, its surface potential remains relatively unchanged after reaching charging saturation once the fabrication process of electret material is determined. Moreover, these increases in charging time and charging voltage have minimal impact on its surface potential. Therefore, when increasing the cathode current/current density after charging saturation by increasing the cathode voltage, it is possible to maintain a stable surface potential for the $SiN_x$-gate. Based on this premise, the $SiN_x$-gate can effectively focus electrons and ensure high electrons transmittance among a wide range of currents/current densities.

**Supplementary Fig. 14 Electrons trajectories of the cathode**

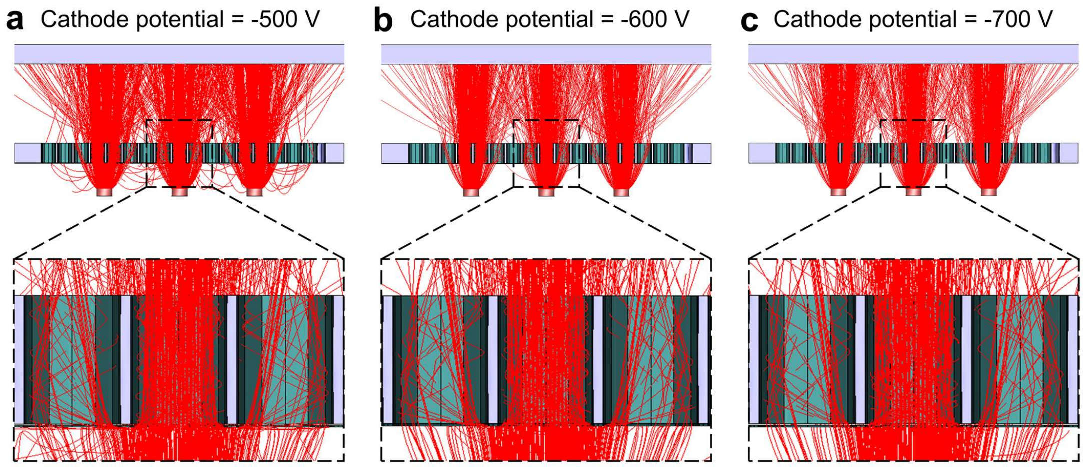


Electrons trajectories at different cathode potentials of (**a**) -500 V, (**b**) -600 V, and (**c**) -700 V when $SiN_x$-gate potential remains constant.

**Supplementary Note 13: The Fowler-Nordheim plots of the cathode.**

Supplementary Fig. 15 shows the Fowler-Nordheim (F-N) plots which can be calculated based on the measured emission currents, revealing that the electron emission from the CNTs emitter follows the features of quantum tunneling behavior.

**Supplementary Fig. 15 The Fowler-Nordheim plots of the cathode**

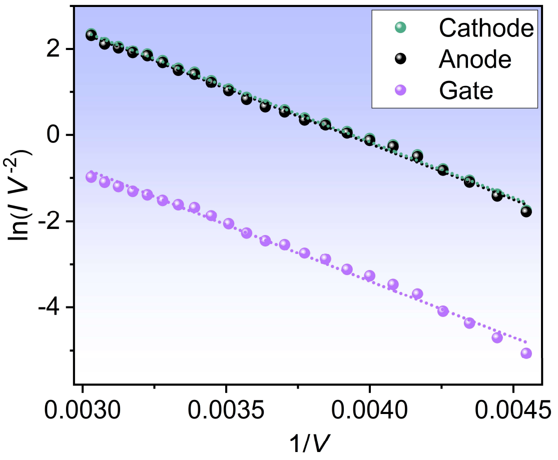


*I* and *V* represent the current and emitter voltage, respectively. The green, black and purple dots correspond to the calculated Fowler-Nordheim (F-N) plots based on measured cathode current, anode current and gate current, respectively. The green, black and purple dotted line correspond to the fitted F-N plots based on measured cathode current, anode current and gate current, respectively.

**Supplementary Note 14: The effect of $SiN_x$ thickness on transmittance of cathode.**

The surface charging of electret materials is manifested through the increase in their surface potential, and there have been a significant number of researches on the relationship between the surface potential of electrets and their thickness. Researchers have also derived the expression for the surface potential of electret materials after charging (Supplementary Equation 1.3)[26, 28]. In the expression, $V_s$ represents the surface potential, $t$ is the thickness of the electret, $\hat{\sigma}$ stands for the average charge density, $\varepsilon_o$ and $\varepsilon_e$ represent the relative permittivity of the vacuum and the electret material, respectively. It's evident from the formula that the surface potential of the electret material is positively correlated with its thickness, and the relationship has been confirmed by the research conducted in previous work[26].

$$V_s = t\hat{\sigma} / \varepsilon_o \varepsilon_e \qquad 1.3$$

However, in practical charging processes, the penetration depth of electrons within the electret material is limited, depending on the initial energy of the electrons. The findings of Z. Gan et al.'s research indicate that when the energy of an E-beam is less than 1 keV, the electron penetration depth within $Si_3N_4$ is below 500 nm[29]. Similarly, the outcomes of V. Leonov et al.'s research demonstrate that the average charging distance of electrons within $Si_3N_4$ is less than 935 nm under a specific voltage[27].

Based on this analysis, the $SiN_x$ electret fabricated in this study also exhibits a maximum electron charging distance (penetration depth) under a specific charging voltage. To further investigate the relationship between surface charging and the thickness of $SiN_x$ electrets, we deposited $SiN_x$ electret layers of varying thicknesses (50 nm, 200 nm, 500 nm, 1000 nm, and 1500 nm) on the Au/Si substrate. After reaching charging saturation under a fixed voltage, we measured the relationship between the surface potential and thickness of the $SiN_x$ electrets. As depicted in Supplementary Fig. 16a, with increasing thickness, the surface potentials of the $SiN_x$ electrets gradually increase. When the thickness of the $SiN_x$ electret reaches 500 nm, further increases in thickness result in minimal changes in surface potential, indicating that the electron penetration depth within the $SiN_x$ electret has reached its maximum at this point (as

shown in the inset in Supplementary Fig. 16a).

**Supplementary Fig. 16 The $SiN_x$ thickness effect on the transmittance**

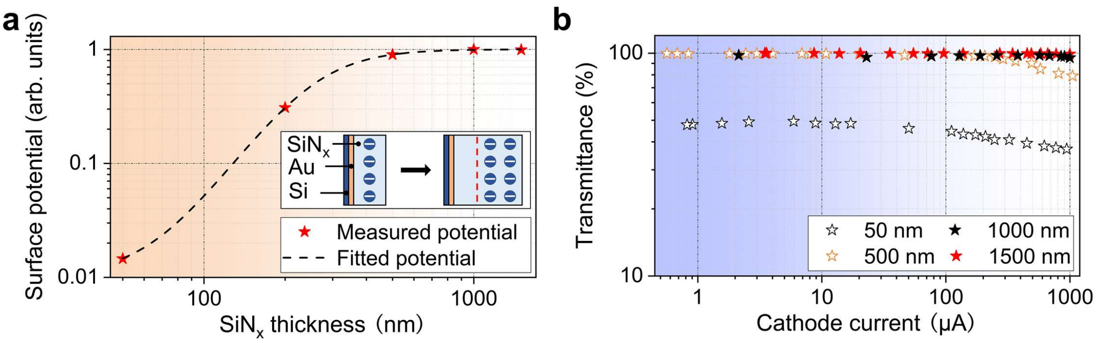


**a** The relationship between normalized surface potential of $SiN_x$ electret and $SiN_x$ thickness, the inset is the schematic of the electron penetration depth within the $SiN_x$ electret. **b** The correlation between electron beam (E-beam) transmittance and $SiN_x$ thickness. The black and orange hollow five-pointed stars represent the measured E-beam transmittances when the $SiN_x$ electret thickness is 50 nm and 500 nm, respectively. The black and red solid five-pointed stars represent the measured E-beam transmittances when the $SiN_x$ electret thickness is 1000 nm and 1500 nm, respectively.

During the operation of a cathode, the magnitude of the surface potential on the $SiN_x$-gate will influence the focusing effect on the E-beam and subsequently impact the E-beam transmittance of the cathode. To further investigate the relationship between $SiN_x$ thickness and E-beam transmittance, we fabricated gates with different $SiN_x$ electret thicknesses, specifically 50 nm, 500 nm, 1000 nm, and 1500 nm, and measured the E-beam transmittance of the cathode in the range of approximately 0-1 mA (corresponding to the current density of 0-17.54 mA $cm^{-2}$). The test results, shown in Supplementary Fig. 16b, reveal that with a thickness of 50 nm, the focusing effect of the E-beam by the $SiN_x$-gate is limited due to the relatively small surface potential of the electret (Supplementary Fig. 16a), resulting in an average E-beam transmittance of approximately 44.28%. As the thickness increases to 500 nm, the surface potential of the $SiN_x$ electret becomes sufficiently large (Supplementary Fig. 16a), implying that the electron charging distance is approaching its maximum value. Consequently, the

average E-beam transmittance for the 500 nm $SiN_x$-gate reaches 95.58%.

With further increases in $SiN_x$ thickness (>500 nm), the average E-beam transmittance of the cathode saturates at >96%. It's important to note that for $SiN_x$ thicknesses below 1000 nm, a slight attenuation in transmittance occurs at high currents (>100 μA) for the cathode. This situation could potentially be attributed to two reasons. Firstly, this could be attributed to the increased initial energy of electrons (due to higher cathode voltage) at high currents, leading to greater electron penetration depth within the $SiN_x$, and consequently causing a small portion of electron loss[29]. Secondly, when using PECVD to deposit thinner $SiN_x$ films on both the surface and sidewalls of the gate, the consistency of the deposited $SiN_x$ thickness on the gate sidewalls could be compromised. This inconsistency might lead to a reduced focusing effect on the gate sidewalls at high currents, which could result in the observed behavior. Therefore, when the cathode emission current falls within the range of approximately 0-1 mA in this study (the corresponding cathode voltage should be less than -700 V), the thickness of the $SiN_x$ electret should be 1 μm or greater.

**Supplementary Note 15: Long-time measurement of the cathode.**

The time dependence of the applied voltage at a constant emission current of 27 μA is shown in Supplementary Fig. 17, illustrating the long-term stability of the system. The current was kept constant by continually applying a direct-current (DC) voltage feedback loop for 550 hours, with the initial value of $V_c$ set at 517.2 V. After the aging process, the average applied $V_c$ is 412.0 V with a fluctuation of 13%, and an average decrease in voltage of 56.4 mV $hr^{-1}$ over time. This phenomenon is attributed to the vacuum pressure within the chamber. At the start of the stability measurement, the vacuum quality is expected to decrease somewhat due to outgassing during the aging process, resulting in a higher voltage supply. As the outgassing process continues, the vacuum condition improves, leading to improved emission performance[30].

**Supplementary Fig. 17 The applied emitter voltage as a function of time**

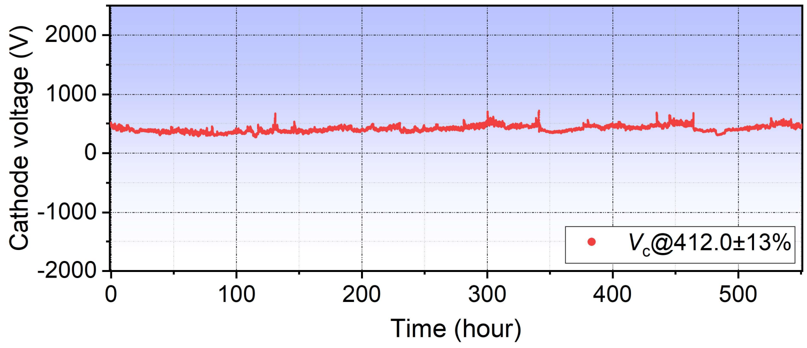


The applied cathode emitter voltage ($V_c$) as a function of time.

**Supplementary Note 16: The high current stability of the cathode.**

To verify the long-term stability of CNTs cathode based on $SiN_x$-gate under a high current level (~mA), we conducted an enduring stability test on cathode emission at around ~3 mA. As illustrated in Supplementary Fig. 18a, over a continuous 60 hours FE test, the cathode maintains an average emission current of 3.25 mA (corresponding to the emitter area of 0.342 $cm^2$ and current density of 9.50 mA $cm^{-2}$), with a current fluctuation of 0.49%. The average E-beam transmittance through the $SiN_x$-gate is measured at 95.30%, with a transmittance fluctuation of 0.30% (Supplementary Fig. 18b). The test outcomes demonstrate that even under high current (3.25 mA) and current density (9.50 mA $cm^{-2}$), the $SiN_x$-gate can sustain its charging characteristics and focusing effects, enabling the achievement of a long-term stable and high E-beam transmittance for mA-level CNTs cathode.

However, further improvement of current density upon this foundation (9.50 mA $cm^{-2}$) may exert influences on the cathode's long-term stability. This is because, under higher current density conditions, the instability of the CNTs emitters, such as carbon evaporation and adsorption resulting from thermal effects[31], could potentially affect the performance of the $SiN_x$-gate, consequently impacting the E-beam transmittance of the cathode. Therefore, to enhance the cathode's emission current range while maintaining a high E-beam transmittance, the focus should be on increasing the emission area of the CNTs rather than the current density, resulting from a larger emitting area can generate a higher current level.

**Supplementary Fig. 18 Long-term currents measurement of the cathode**

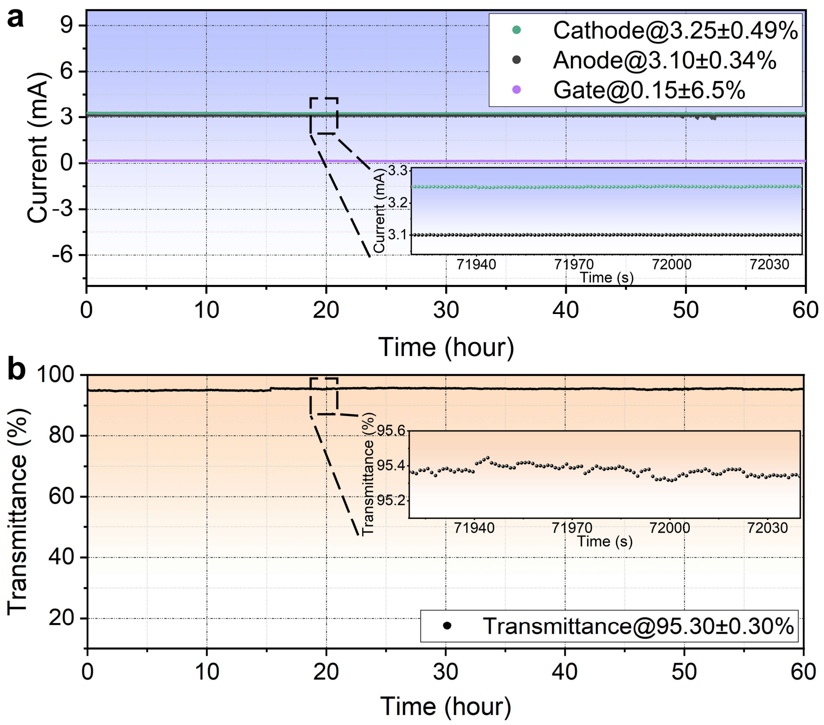


**a** Emission current stability of the cathode at 3.25 mA, the inset illustrates the current fluctuations of cathode current and anode current within 2 minutes. The green, black and purple dots correspond to the measured cathode current, anode current and gate current, respectively. **b** Transmittance of the cathode, the inset is the electron beam (E-beam) transmittance fluctuation within 2 minutes. The emitter area is 0.342 cm$^2$. The black dot represents the measured E-beam transmittance based on $SiN_x$-gate.

**Supplementary Note 17: The cathode performances upon the external stimuli.**

The charging performance of $SiN_x$ may be influenced by external stimuli, thereby affecting the performances of the cathode. Therefore, we further analyzed parameters that could potentially impact the charging performance of $SiN_x$ shown in Supplementary Table 2, such as environmental vibrations, thermal energy, light energy, electrical energy, etc.

**Supplementary Table 2 Parameters affecting the charging performance of $SiN_x$**

| Parameter | Influencing mechanism | Reference |
| --- | --- | --- |
| Mechanical force and temperature cycling | Film degradation | [32] |
| Thermal energy | Thermal-stimulated discharge | [33, 34] |
| Light energy | Photo-stimulated discharge | [35, 36] |
| Electrical energy | Charge distribution | [37] |

For the application of CNTs cathode in space exploration, ground-based environmental simulation tests are essential. In accordance with aerospace standards, before the launch of the TianQin-1 satellite, a comprehensive ground-based environmental performance verification is required to ensure the system's reliability[32]. This verification process consists of two essential components: mechanical performance testing and temperature performance testing. The mechanical performance tests entail impact and vibration tests, while the temperature performance tests encompass the temperature cycle test at atmospheric pressure and thermal vacuum. Hence, we initially investigated the impact of ground-based environmental simulation tests on the performances of $SiN_x$-gate and cathode.

As depicted in Supplementary Fig. 19a, to facilitate environmental simulation testing, an engineering prototype of the cathode was designed. The upper and lower covers of the prototype were fabricated using PEEK insulation material, while the cathode signal was routed through the underlying circuit board. Finally, Supplementary Fig. 19b illustrates the physical representation of the fabricated engineering prototype of the cathode.

**Supplementary Fig. 19 The design of the engineering prototype of the cathode**

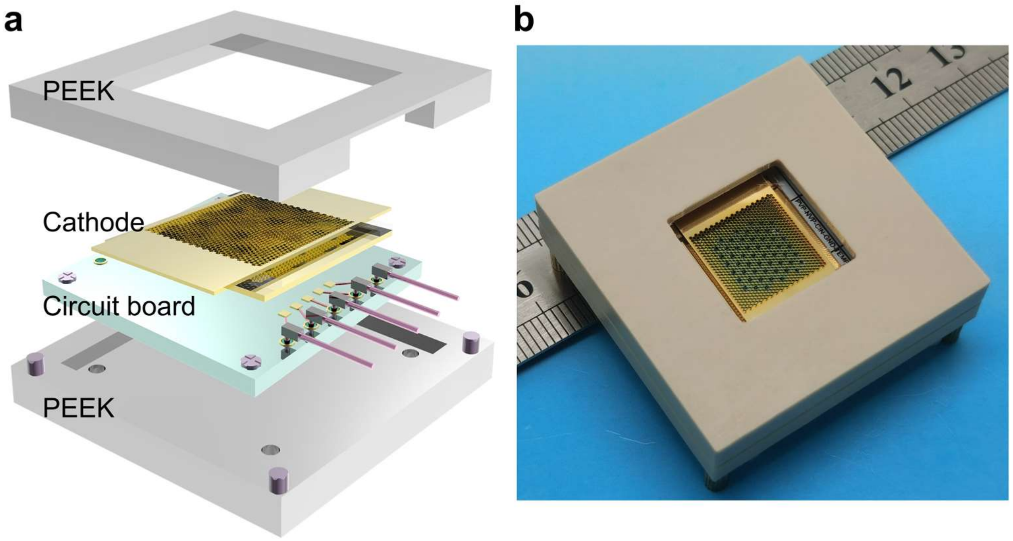


**a** Schematic and (**b**) physical images of the cathode engineering prototype based on carbon nanotubes (CNTs) emitter.

The parameters for the temperature cycle tests at atmospheric pressure and thermal vacuum are presented in Supplementary Table 3.

**Supplementary Table 3 Parameters of the temperature performance tests**

| Parameter | Atmospheric pressure thermal cycling | Vacuum thermal cycling |
|---|---|---|
| Test pressure | Atmospheric pressure | $<6.65\times10^{-3}$ Pa |
| Test temperature | -25 °C-60 °C | -25 °C-60 °C |
| Cycle | 13.5 | 3.5 |
| Warming rate | 3 °C-5 °C $min^{-1}$ | 1 °C-3 °C $min^{-1}$ |

Supplementary Fig. 20a depicts the experimental instrument (JTK-225L-B) utilized in the ambient pressure thermal cycling experiment (left), concurrently illustrating the configuration of the cathode within the instrument (right). Supplementary Fig. 20b illustrates the temperature profile for the atmospheric pressure thermal cycling, it can be observed from the figure that the test curve closely adheres to the specified parameter requirements.

**Supplementary Fig. 20 Atmospheric thermal cycling for the cathode**

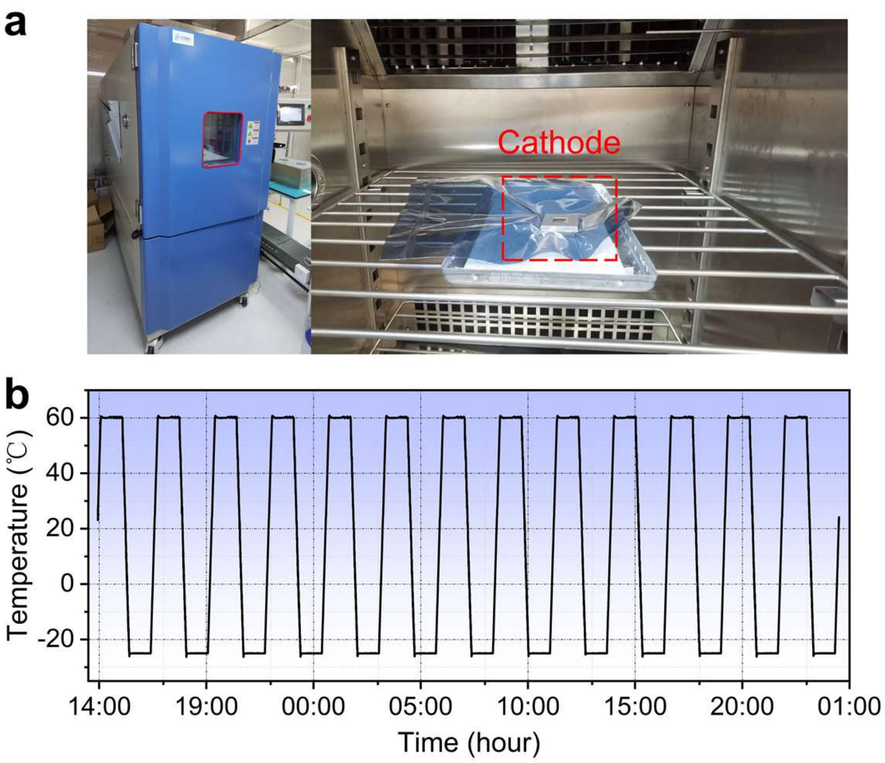


**a** The experimental instrument and (**b**) the test curve for atmospheric thermal cycling of the carbon nanotubes (CNTs) cathode, respectively.

Supplementary Fig. 21a depicts the experimental instrument (KM0812-CPLN) utilized in the vacuum thermal cycling experiment (left), concurrently illustrating the configuration of the cathode within the instrument (right). Supplementary Fig. 21b illustrates the temperature profile for the vacuum thermal cycling, it can be observed from the figure that the test curve closely adheres to the specified parameter requirements. Following the thermal cycling experiments, no significant changes were observed in the morphology of the cathode.

**Supplementary Fig. 21 Vacuum thermal cycling for the cathode**

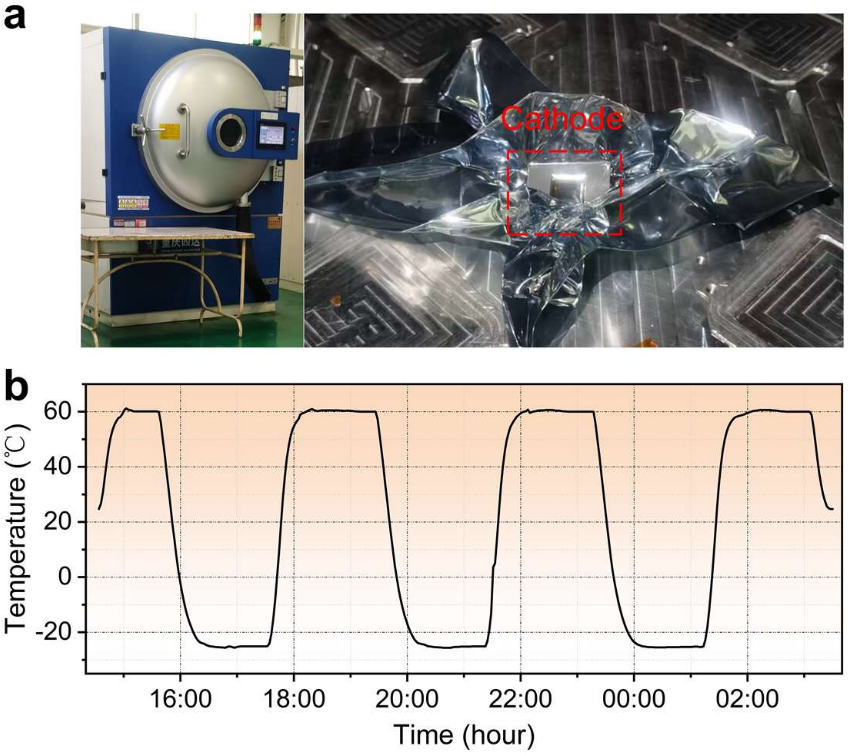


**a** The experimental instrument and (**b**) the test curve for vacuum thermal cycling of the carbon nanotubes (CNTs) cathode, respectively.

After completing the temperature performance tests, we conducted impact tests using SY14A-100 (Supplementary Fig. 22a) to verify the impact resistance of the cathode. The impact test necessitates an impact force of 700 g along each axis. The impact characteristics should exhibit a range of up to 6 dB $oct^{-1}$ between 100 Hz and 600 Hz, and up to 700 g between 600 Hz and 4000 Hz, as depicted in Supplementary Fig. 22b.

**Supplementary Fig. 22 The impact tests for the cathode**

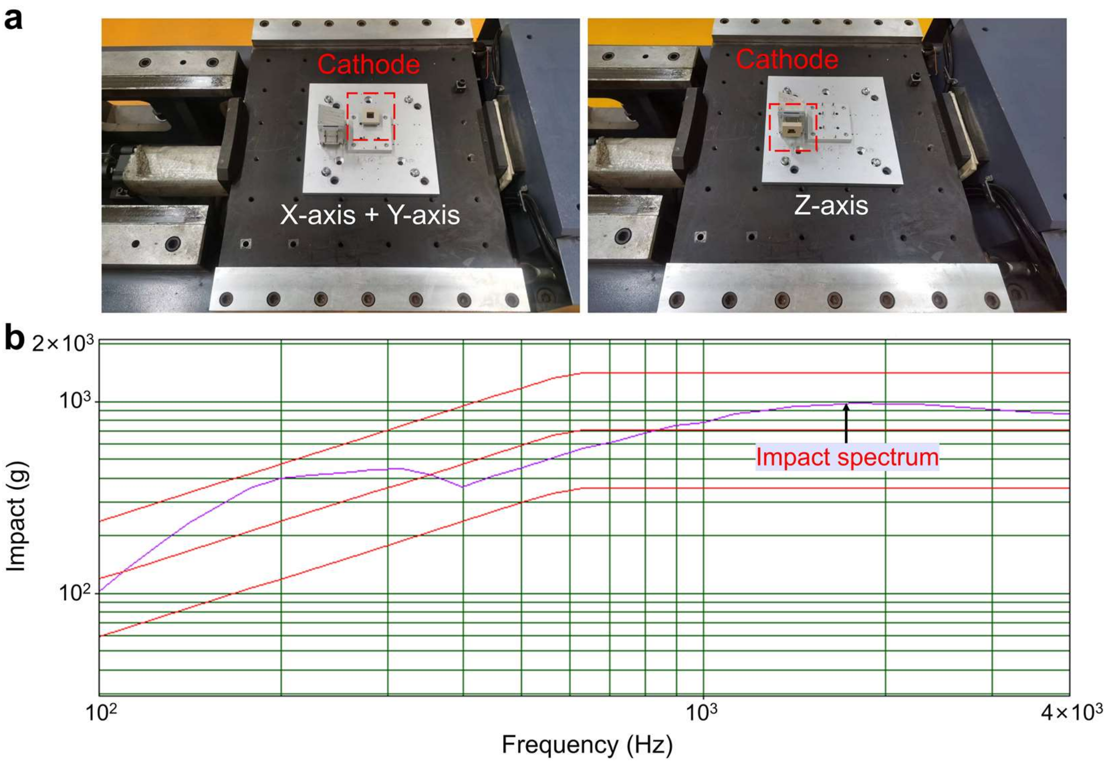


**a** The experimental instrument and (**b**) the test curves for impact tests of the carbon nanotubes (CNTs) cathode, respectively.

In the vibration tests, sinusoidal and random vibrations were applied along the three orthogonal axes using ES-10-240 (Supplementary Fig. 23). The sinusoidal vibration has an amplitude of 10 g within the frequency range of 20 Hz to 100 Hz, with a scan rate of 4 octaves/min shown in Supplementary Fig. 24a-c. The total root mean square of the random vibration is 9.64 g, and the load time is 1 minute. And the power spectral density of the random vibration is depicted in Supplementary Fig. 25a-c. Following the test, we conducted a verification of the electrical connection and surface topography of the cathode prototype. The results confirm that the cathode prototype successfully passed the mechanical performance tests.

**Supplementary Fig. 23 The experimental instrument for vibration tests**

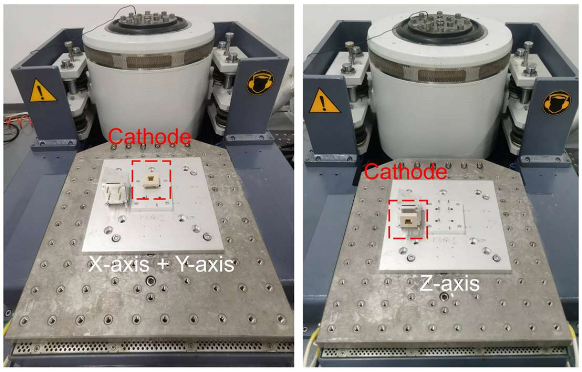


The experimental instrument for vibration tests of the carbon nanotubes (CNTs) cathode.

**Supplementary Fig. 24 The sinusoidal vibration for the cathode**

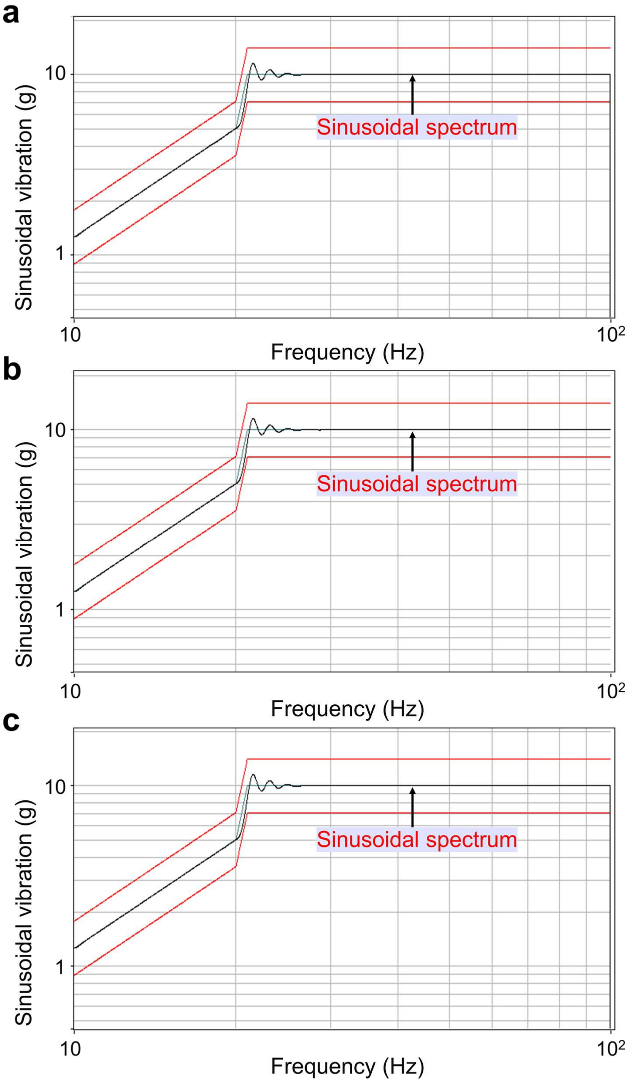


The sinusoidal vibration spectra along the (**a**) X, (**b**) Y, and (**c**) Z axis of carbon nanotubes (CNTs) cathode.

**Supplementary Fig. 25 The random vibration for the cathode**

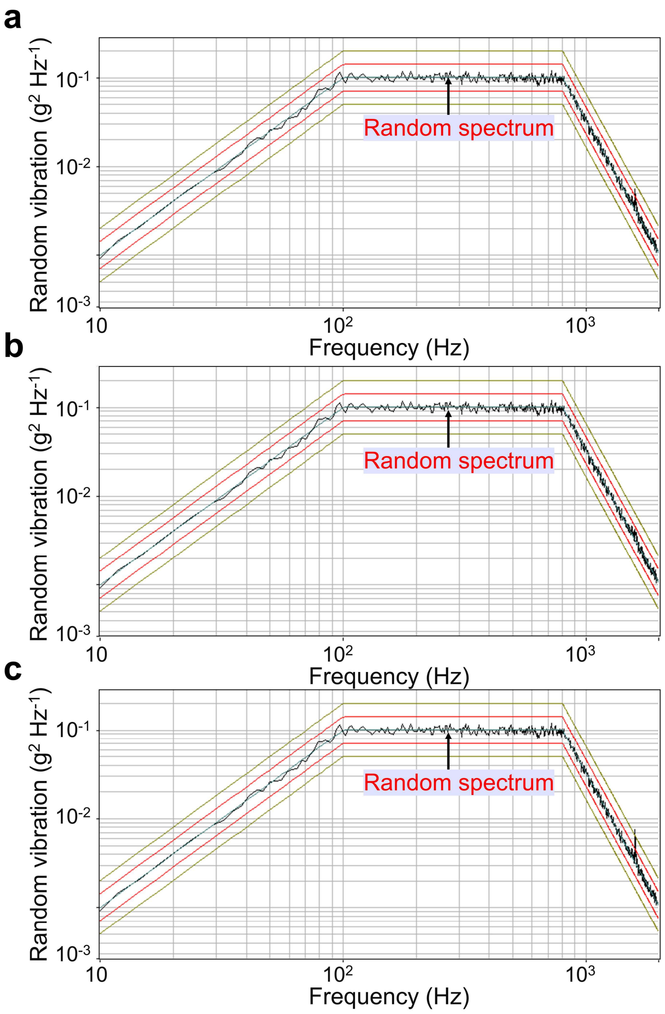


The random vibration spectra along the (**a**) X, (**b**) Y, and (**c**) Z axis of carbon nanotubes (CNTs) cathode.

After conducting the ground-based environmental simulation tests, a comparison was made between the pre-and post-test E-beam transmittance of the cathode. The results clearly demonstrate that there are no significant changes in the transmittance of the cathode (Supplementary Fig. 26). This further supports the robustness and reliability of the cathode and gate under external stimuli.

**Supplementary Fig. 26 The transmittances versus cathode current plots**

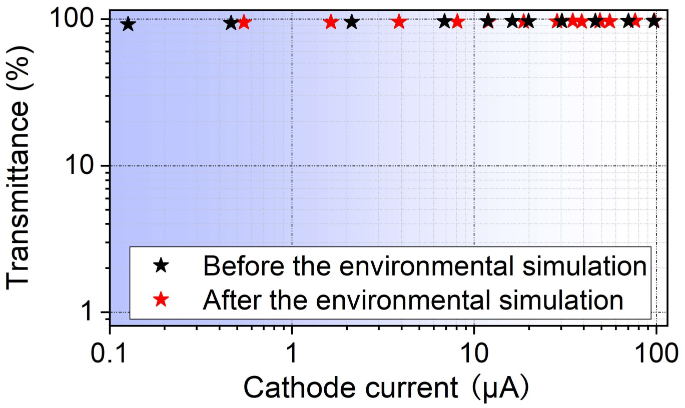


The transmittances versus cathode current plots before and after the environmental simulations. The black and red five-pointed stars represent the measured electron beam (E-beam) transmittances before and after the environmental simulations, respectively.

After reaching saturation upon charging, the internally stored space charges within electret materials are typically in a frozen state[38]. However, when electrets are subjected to heating, the mobility of internal space charges increase rapidly. Consequently, thermal stimulation significantly reduces the decay time of the electret's charges. The positions and shapes of peaks in the current-temperature spectrum generated by thermal-stimulated discharge reflect effectively the microscopic characteristics of stored charges within the electret materials. Extensive research into the thermal-stimulated discharge characteristics of $SiN_x$ electret has been carried out in previous works[33, 34]. According to the findings, thermal stimulation discharge occurs when the $SiN_x$ electret is heated above 300 °C, thereby altering the charge distribution within the electret.

However, in contrast to thermionic cathodes, CNTs-based cathode (named cold cathode) can operate at room temperature[20, 21]. Therefore, the impact of thermal-stimulated discharge on the charging performance of $SiN_x$ is not considered in practical processes. Moreover, during the cathode's actual operational process, electrons produced by CNTs continuously emit onto the $SiN_x$-gate surface. Thus, the $SiN_x$ electret should be in a state of real-time charge saturation. In conclusion, thermal stimulation does not affect the high transmittance characteristics of CNTs cathode.

Additionally, similar to thermal-stimulated discharge, photo-stimulation can induce discharge phenomena in electret materials. A. Mellinger et al. have extensively studied photo-stimulated discharge in electret materials[35, 36]. Under ultraviolet (UV) light irradiation, electrets exhibit a weak discharge phenomenon, affecting the internal charge distribution within the material. Based on this, to investigate the impact of light irradiation on the charging performance of $SiN_x$ electret, we conducted the following experiments illustrated in Supplementary Fig. 27a.

**Supplementary Fig. 27 The photo-stimulated discharge of $SiN_x$ electret**

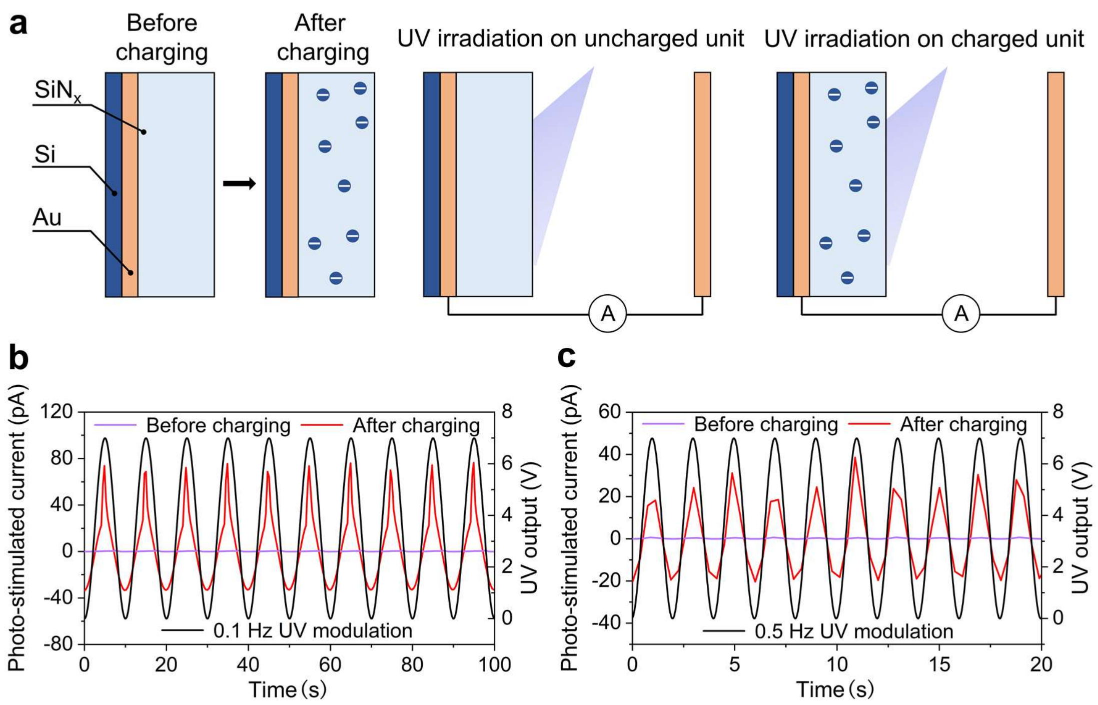


**a** The schematic diagram of ultraviolet (UV) irradiation for the $SiN_x$ electret before and after charging, the currents can be measured using the electrometer (A). The photo-stimulated current under (**b**) 0.1 Hz UV modulation and (**c**) 0.5 Hz UV modulation. The purple and red curves correspond the photo-stimulated current under different modulation frequencies before and after charging, respectively, meanwhile the black curves represent the different UV output.

We utilized the setup to measure the current of the $SiN_x$ electret before and after charging under the irradiation of the UV light source (TUD59H1B, Sensor Electronic Technology, Inc) with peak emission wavelengths from 250 nm to 260 nm. From

Supplementary Fig. 27b-c, it can be observed that when the UV light source is modulated at frequencies of 0.1 Hz and 0.5 Hz, the discharged current of the charged $SiN_x$ reaches several tens of pA, whereas the discharged current of the $SiN_x$ electret before charging remains very small. These test results indicate the presence of weak photo-stimulated discharge in the $SiN_x$ electret.

To investigate the impact of photo-stimulated discharge on the transmittance of CNTs cathode, we employed the setup depicted in Supplementary Fig. 28a to measure the E-beam transmittances of the cathode under both UV light irradiation and no UV light irradiation conditions. The test results presented in Supplementary Fig. 28b clearly indicate that UV irradiation has minimal effect on the E-beam transmittance of the cathode. Across the emission current range of 0-100 μA, the E-beam transmittances of the cathode remain consistently above 96% regardless of whether UV irradiation is present or not.

**Supplementary Fig. 28 The transmittance of cathode under ultraviolet irradiation**

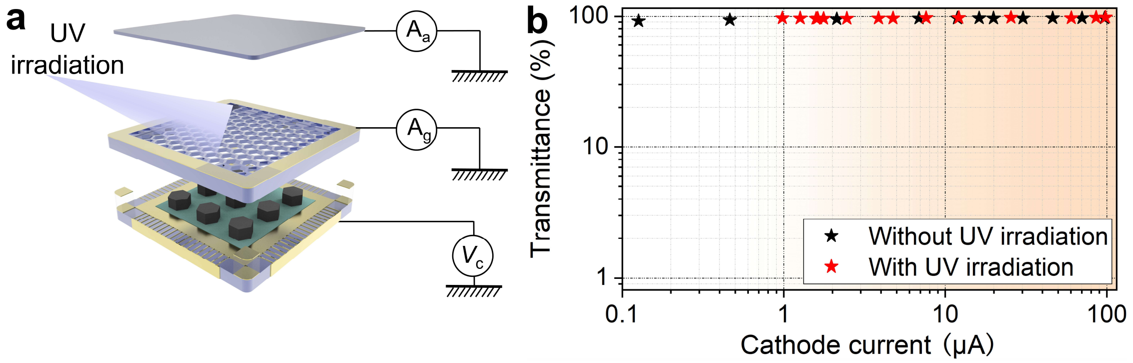


**a** The illustration for cathode testing under ultraviolet (UV) irradiation, the currents of anode and gate can be measured using the electrometer ($A_a$ and $A_g$) under applied emitter voltage ($V_c$). **b** The transmittance versus cathode current plots with and without UV irradiation. The black and red five-pointed stars represent the measured electron beam (E-beam) transmittances without and with UV irradiation, respectively.

Furthermore, we investigated the short-term stability of E-beam transmittances in CNTs cathode under different UV light modulation frequencies. These modulation frequencies are set at 0 Hz, 0.1 Hz, 1 Hz, and 10 Hz. At an emitted cathode current of

10 μA, E-beam transmittances consistently exceed 96% for all UV modulation frequencies, with fluctuations of less than 0.3% (Supplementary Fig. 29a-d).

**Supplementary Fig. 29 The transmittance of the cathode under ultraviolet stimuli**

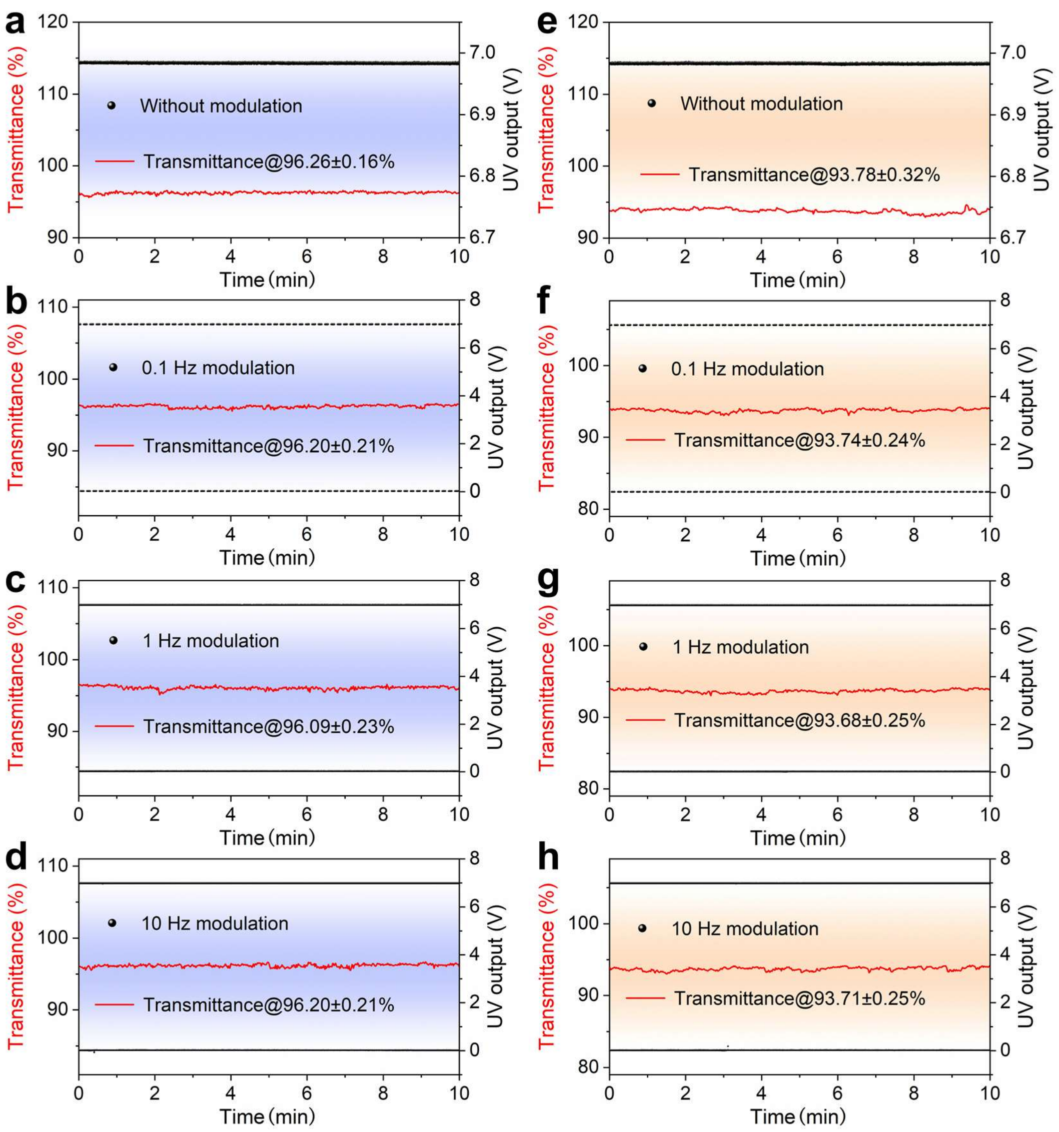


The transmittances of the cathode under different ultraviolet (UV) modulations of (**a**) 0 Hz, (**b**) 0.1 Hz, (**c**) 1 Hz, and (**d**) 10 Hz when the cathode current is ~10 μA. The transmittances of the cathode under different UV modulations of (**e**) 0 Hz, (**f**) 0.1 Hz, (**g**) 1 Hz, and (**h**) 10 Hz when the cathode current is ~100 μA. The black dots correspond the UV output and red curves represent the electron beam (E-beam) transmittance under different UV modulation frequencies.

Similarly, when the emitted cathode current is increased to 100 μA, E-beam

transmittances remain above 93% for various UV modulation frequencies, accompanied by transmittance fluctuations of less than 0.4% (Supplementary Fig. 29e-h). Experimental outcomes further indicate the negligible impact of photo-stimulation on the E-beam transmittance of CNTs cathode.

In addition to thermal and photo-stimulated discharge, electrical stimulation could also potentially impact the charging performance of $SiN_x$, and hence we investigated the variations in E-beam transmittance under different current steps and modulation frequencies. As illustrated in Supplementary Fig. 30a-b, a 0.1 Hz current modulation was used to check the charging stability of the $SiN_x$ electret under different current steps (corresponding to different voltage steps).

**Supplementary Fig. 30 The transmittances of the cathode under electrical stimuli**

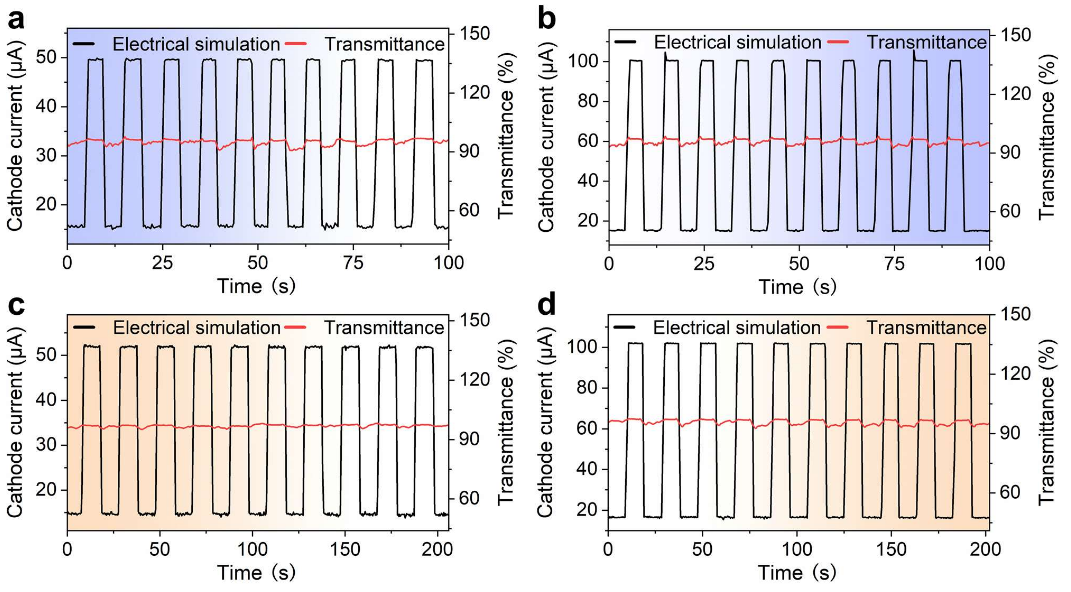


The transmittances of the cathode during transitions of the cathode current from (**a**) ~10 μA to ~50 μA and from (**b**) ~10 μA to ~100 μA under 0.1 Hz current modulation. The transmittances of the cathode during transitions of the cathode current from (**c**) ~10 μA to ~50 μA and from (**d**) ~10 μA to ~100 μA under 0.05 Hz current modulation. The black and red curves represent the electrical simulation and corresponding electron beam (E-beam) transmittance, respectively.

Over a series of 10 current modulation cycles ranging from ~10 μA to ~50 μA, the

average E-beam transmittance of the $SiN_x$-gate remains consistently at 94.98%, exhibiting a low fluctuation of only 1.6%. In a sequence of 10 cycles during which the current undergoes a stepwise increase from ~10 μA to ~100 μA, the mean E-beam transmittance across the $SiN_x$-gate electrode persists at 95.74%, exhibiting a marginal variability of only 1.5%.

At the modulation frequency of 0.05 Hz, during transitions of the cathode current from ~10 μA to ~50 μA (Supplementary Fig. 30c) and from ~10 μA to ~100 μA (Supplementary Fig. 30d), the average E-beam transmittances across the $SiN_x$-gate electrode are measured at 96.89% and 95.74%, with corresponding transmittance fluctuations of 0.70% and 1.4%, respectively. All test results consistently demonstrate that despite slight fluctuations in the E-beam transmittance of the $SiN_x$-gate electrode during electrical stimulation, it can be still maintained at relatively high levels of transmittance. Therefore, the influence of electrical stimulation on the charging stability of $SiN_x$ is minimal.

**Supplementary Note 18: The long-term current stability of the cathode.**

Following the investigations into the influence of external stimulation on cathode performance outlined in Supplementary Table 2, a long-term test of 310 hours was conducted on the CNTs cathode to verify its enduring stability. As depicted in Supplementary Fig. 31a, the average emitted current over the course of the cathode's enduring testing is 26.35 μA, with a current fluctuation of merely 1.9%. The average E-beam transmittance remains at 97.79% with a fluctuation of 0.89% (Supplementary Fig. 31b). These results collectively highlight the minimal impact of external stimulation on cathode performances, further validating the reliability of our structural design and the stability of $SiN_x$'s charging performance.

**Supplementary Fig. 31 Long-term current measurements of the cathode**

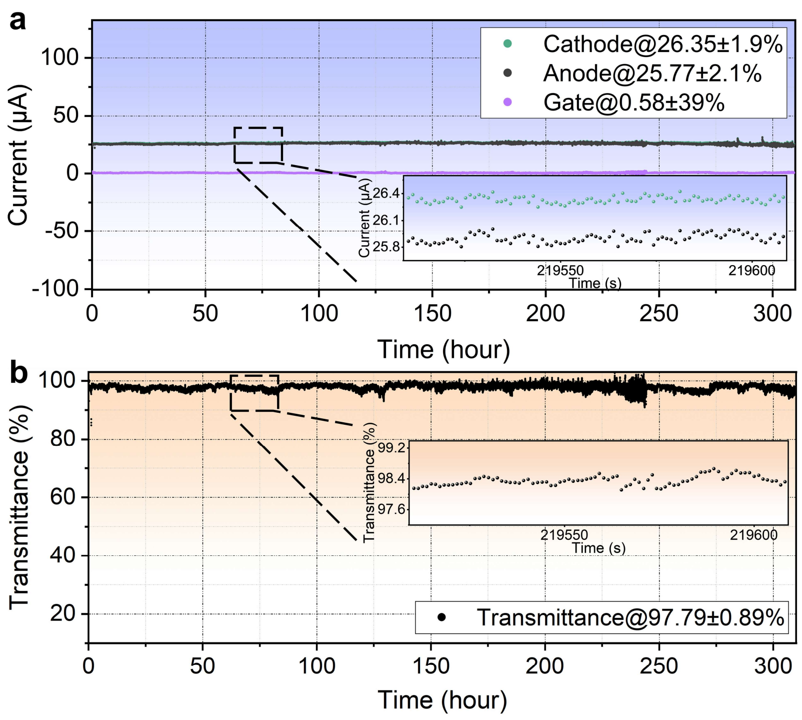


**a** Emission current stability of the cathode at 26.35 μA, the inset illustrates the current fluctuations of cathode current and anode current within 100 s. The green, black and purple dots correspond to the measured cathode current, anode current and gate current, respectively. **b** Transmittance of the cathode, the inset is the electron beam (E-beam) transmittance fluctuation within 100 s. The emitter area is 0.057 $cm^2$. The black dot represents the measured E-beam transmittance based on $SiN_x$-gate.

**Supplementary References**

1. Zaghloul, U.*,* et al. Effect of deposition gas ratio, RF power, and substrate temperature on the charging/discharging processes in PECVD silicon nitride films for electrostatic NEMS/MEMS reliability using atomic force microscopy. *JMEMS* **20**, 1395-1418 (2011).

2. Birmpiliotis, D., Stavrinidis, G., Koutsoureli, M., Konstantinidis, G., Papaioannou, G. On the discharge transport mechanisms through the dielectric film in MEMS capacitive switches. *JMEMS* **29**, 202-213 (2020).

3. Malik, P., Gupta, H., Ghosh, S., Srivastava, P. Study of optical properties of single and double layered amorphous silicon nitride films for photovoltaics applications. *Silicon*. **15**, 143-151 (2022).

4. Fujii, S.*,* et al. Efficient field emission from an individual aligned carbon nanotube bundle enhanced by edge effect. *Appl*. *Phys*. *Lett*. **90**, 153108 (2007).

5. Shiffler, D.*,* et al. Emission uniformity and emission area of explosive field emission cathodes. *Appl*. *Phys*. *Lett*. **79**, 2871-2873 (2001).

6. Cole, M. T., Teo, K. B., Groening, O., Gangloff, L., Legagneux, P., Milne, W. I. Deterministic cold cathode electron emission from carbon nanofibre arrays. *Sci*. *Rep*. **4**, 1-5 (2014).

7. Tang, W., Shiffler, D., Golby, K., LaCour, M., Knowles, T. Experimental study of electric field screening by the proximity of two carbon fiber cathodes. *J*. *Vac*. *Sci*. *Technol*. **30**, 061803 (2012).

8. Harris, J. R., Jensen, K. L., Tang, W., Shiffler, D. A. Control of bulk and edge

screening effects in two-dimensional arrays of ungated field emitters. *J. Vac. Sci. Technol. B* **34**, 041215 (2016).

9. Rezinkina, M. Mathematical modelling of the electric field of carbon nanotube arrays used in cold cathode electron emission devices. *J. Electrostat.* **109**, 103544 (2021).

10. Harris, J. R., Jensen, K. L., Shiffler, D. A. Dependence of optimal spacing on applied field in ungated field emitter arrays. *AIP Adv.* **5**, 087182 (2015).

11. Harris, J. R., Jensen, K. L., Shiffler, D. A., Petillo, J. J. Shielding in ungated field emitter arrays. *Appl. Phys. Lett.* **106**, 201603 (2015).

12. Harris, J. R., Jensen, K. L., Shiffler, D. A. Modelling field emitter arrays using line charge distributions. *J. Phys. D: Appl. Phys.* **48**, 385203 (2015).

13. Guo, Z., Patil, Y., Shinohara, A., Nagura, K., Yoshida, M., Nakanishi, T. Organic molecular and polymeric electrets toward soft electronics. *Mol. Syst. Des. Eng.* **7**, 537-552 (2022).

14. Asher, J., Huang, Z., Cui, C., Wang, J. Multi-scale modeling of ionic electrospray emission. *J. Appl. Phys.* **131**, 014902 (2022).

15. Parmar, S. M., Collins, A. L., Wirz, R. E. Electrospray plume modeling for rapid life and performance analysis. *AIAA SCITECH 2022 Forum.* **20**, 1395-1418 (2022).

16. Kleshch, V. I., Ismagilov, R. R., Mukhin, V. V., Orekhov, A. S., Filatyev, A. S., Obraztsov, A. N. Nano-graphite field-emission cathode for space electric propulsion systems. *Nanotechnol.* **33**, 415201 (2022).

17. Robertson, J. Electronic structure of silicon nitride. *Philos. Mag. B* **63**, 47-77 (2006).

18. Robertson, J., Powell, M. J. Gap states in silicon nitride. *Appl. Phys. Lett.* **44**, 415-

417 (1984).

19. Picciotto, A., Bagolini, A., Bellutti, P., Boscardin, M. Influence of interfaces density and thermal processes on mechanical stress of PECVD silicon nitride. *Appl. Surf. Sci.* **256**, 251-255 (2009).

20. Kang, J. S., Hong, J. H., Park, K. C. High-performance carbon-nanotube-based cold cathode electron beam with low-thermal-expansion gate electrode. *J. Vac. Sci. Technol. B* **36**, 02C104 (2018).

21. Yuan, X.*,* et al. A gridded high-compression-ratio carbon nanotube cold cathode electron gun. *IEEE Electron Device Lett.* **36**, 399-401 (2015).

22. Zaghloul, U., Bhushan, B., Pons, P., Papaioannou, G. J., Coccetti, F., Plana, R. On the influence of environment gases, relative humidity and gas purification on dielectric charging/discharging processes in electrostatically driven MEMS/NEMS devices. *Nanotechnol.* **22**, 035705 (2011).

23. Plopeanu, M. C., Notingher, P. V., Dumitran, L. M., Tabti, B., Antoniu, A., Dascalescu, L. Surface potential decay characterization of non–woven electret filter media. *IEEE Trans. Dielectr. Electr. Insul.* **18**, 1393-1400 (2011).

24. Cui, L. L.*,* et al. The comparative studies of charge storage stabilities among three PP/porous PTFE/PP electret. *J. Electrostat.* **67**, 412-416 (2009).

25. Amjadi, H., Thielemann, C. Silicon-based inorganic electrets for application in micromachined devices. *IEEE Trans. Dielectr. Electr. Insul.* **3**, 494-498 (1996).

26. Zou, X., Zhang, J. Study on PECVD $SiO_2/Si_3N_4$ double-layer electrets with different thicknesses. *Sci. China Technol. Sci.* **54**, 2123-2129 (2011).

27. Leonov, V., Hoof, C. V., Goedbloed, M., Schaijk, R. V. Charge injection and storage in single-layer and multilayer inorganic electrets based on $SiO_2$ and $Si_3N_4$. *IEEE Trans. Dielectr. Electr. Insul.* **19**, 1253-1260 (2012).

28. Wu, M. L., Wang, D., Wan, L. J. Directed block copolymer self-assembly implemented via surface-embedded electrets. *Nat. Commun.* **7**, 10752 (2016).

29. Zhenghao, G., Cher Ming, T., Guan, Z. Nondestructive void size determination in copper metallization under passivation. *IEEE Trans. Device Mater. Reliab.* **3**, 69-78 (2003).

30. Kato, H., O'Rourke, B. E., Suzuki, R. Stable and high current density electron emission using coniferous carbon nano-structured emitter. *Diam. Relat. Mater.* **55**, 41-44 (2015).

31. Williams, L. T., Kumsomboone, V. S., Ready, W. J., Walker, M. L. R. Lifetime and failure mechanisms of an arrayed carbon nanotube field emission cathode. *IEEE Trans. Electron Devices* **57**, 3163-3168 (2010).

32. Li, Z., et al. Design and performance test of the spaceborne laser in the Tianqin-1 mission. *Opt. Laser Technol.* **141**, 107155 (2021).

33. Amjadi, H., Sessler, G. M. Charge storage in APCVD silicon nitride. *IEEE CEIDP Annual Report* **1**, 64-67 (1997).

34. Kressmann, R., Sessler, G. M., Gunther, P. Space-charge electrets. *IEEE Trans. Dielectr. Electr. Insul.* **3**, 607-623 (1996).

35. Mellinger, A., Gonzalez, F. C., Gerhard-Multhaupt, R. Photostimulated discharge in electret polymers: An alternative approach for investigating deep traps. *IEEE Trans.*

*Dielectr*. *Electr*. *Insul*. **11**, 218-226 (2004).

36. Mellinger, A., Gonzalez, F. C., Gerhard-Multhaupt, R., Santos, L. F., Faria, R. M. Photostimulated discharge of corona and electron-beam charged electret polymers. *IEEE ISDE Proceedings* (2002).

37. Papaioannou, G., Coccetti, F., Plana, R. On the modeling of dielectric charging in RF-MEMS capacitive switches. *IEEE SiRF* 108-111 (2010).

38. Turnhout, J. V. Thermally stimulated discharge of electrets. *Springer* (2005).
